# Integrated multi-omics identifies pathways governing interspecies interaction between *A. fumigatus* and *K. pneumoniae*

**DOI:** 10.1038/s42003-024-07145-x

**Published:** 2024-11-12

**Authors:** Tamires Bitencourt, Filomena Nogueira, Sabrina Jenull, Trinh Phan-Canh, Michael Tscherner, Karl Kuchler, Thomas Lion

**Affiliations:** 1 CCRI – St. Anna Children’s Cancer Research Institute, Vienna, Austria; 2 Labdia - Labordiagnostik GmbH, Vienna, Austria; 3 Department of Medical Biochemistry, Campus Vienna Biocenter, Max Perutz Labs, Medical University of Vienna, Vienna, Austria; 4 Department of Pathobiology, Institute of Microbiology, University of Veterinary Medicine Vienna, Vienna, Austria; 5 Department of Pediatrics, Medical University of Vienna, Vienna, Austria

**Keywords:** Biofilms, Infection, Diagnostic markers

## Abstract

Polymicrobial co- and superinfections involving bacterial and fungal pathogens pose serious challenges for diagnosis and therapy, and are associated with elevated morbidity and mortality. However, the metabolic dynamics of bacterial–fungal interactions (BFI) and the resulting impact on disease outcome remain largely unknown. The fungus *Aspergillus fumigatus* and the bacterium *Klebsiella pneumoniae* are clinically important pathogens sharing common niches in the human body, especially in the lower respiratory tract. We have exploited an integrated multi-omics approach to unravel the complex and multifaceted processes implicated in the interspecies communication involving these pathogens in mixed biofilms. In this setting, *A. fumigatus* responds to the bacterial challenge by rewiring its metabolism, attenuating the translational machineries, and by connecting secondary with primary metabolism, while *K. pneumoniae* maintains its central metabolism and translation activity. The flexibility in the metabolism of *A. fumigatus* and the ability to quickly adapt to the changing microenvironment mediated by the bacteria highlight new possibilities for studying the impact of cross-communication between competing interaction partners. The data underscore the complexity governing the dynamics underlying BFI, such as pronounced metabolic changes mounted in *A. fumigatus* interacting with *K. pneumoniae*. Our findings identify candidate biomarkers potentially exploitable for improved clinical management of BFI.

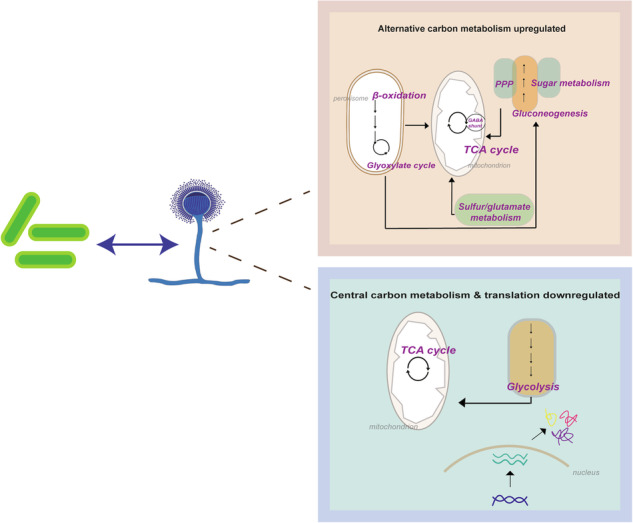

## Abstract (web summary)

A multi-omics study highlights the flexibility in the metabolism of *Aspergillus fumigatus* in microenvironments co-shared with *Klebsiella pneumoniae*, uncovering potential biomarkers to enhance clinical care for polymicrobial infections.

## Introduction

Invasive fungal infections show high mortality rates, claiming globally more than 1.5 million lives per year^1,2^. In this worldwide scenario, *A. fumigatus* (Afu) is a prominent fungal pathogen, responsible for about 300,000 cases of invasive aspergillosis per year. In the immunocompromised setting, the reported lethality of Afu infections ranges from 50% to 100%, depending on the timeliness of diagnosis and the adequacy of treatment^2,3^. The WHO therefore ranks Afu as a critical fungal pathogen requiring the highest priority for research and drug discovery^4^.

Despite this staggering impact on human health, many aspects related to the pathophysiology of fungal infections are ill-defined. Although many studies have focused on unveiling molecular determinants associated with *A. fumigatus* infections, the complexity of processes underlying the metabolic flexibility of this pathogen remains largely unknown^5^. The most likely multifactorial processes involved in Afu interactions with other microbiota at various sites, prominently involving the lungs as well as the host immune surveillance, have an important impact on clinical outcome^6–8^. The spatial arrangements and exchanges upon polymicrobial interactions are determining factors for the regulation of host microenvironment dynamics affecting nutrient availability, distribution of microbial symbionts and immune responses^9^. Hence, a detailed understanding of the interplay between bacterial and fungal pathogens might uncover important clinical aspects of polymicrobial infections relevant for diagnosis and treatment. Indeed, polymicrobial infections involving bacterial–fungal interactions (BFIs) pose serious therapeutic challenges, since BFIs are associated with challenging clinical manifestations such as increased antimicrobial drug resistance or immune evasion, and thus with higher morbidity and mortality^10^.

Importantly, *Klebsiella pneumoniae* (*K. pneumoniae*), which belongs to the antimicrobial-resistant ESKAPE pathogens (***E****nteroccocus faecium*, ***S****taphylococcus aureus*, ***K****. pneumoniae*, ***A****cinetobacter baumannii*, ***P****seudomonas aeruginosa* and ***E****nterobacter species*), has been associated with a significant number of community-acquired and nosocomial infections, resulting in high morbidity and mortality. Infections caused by the ESKAPE pathogens have sparked great concerns owing to the increased disease burden, failure of treatment and elevated death rates^11–13^. Due to the global human health threat represented by the ESKAPE pathogens, the WHO included them in the critical priority group for antibiotic research and development^14^. In the context of BFI, *Klebsiella* strains present in mixed biofilm communities are often hypervirulent^15^.

Hence, both *A. fumigatus* and *K. pneumoniae* are clinically highly relevant pathogens sharing common niches in the body, including particularly the lower respiratory tract, which represents a site of their potential interactions. These interactions appear to be largely antagonistic, as *K. pneumoniae* is capable of inhibiting the growth of *A. fumigatus*^16,17^. Indeed, previous studies showed inhibitory effects of *K. pneumoniae* on Afu germination and hyphae formation upon contact with live bacteria and a significant fungal biomass reduction upon exposure to bacterial filtrates^16,17^. However, the metabolic networks and pathways driving this interaction remain unresolved. It is unclear how antagonistic BFIs reduce the fungal burden, but the reduced fungal loads persist in long co-cultures and in contexts of chronic infection, maintaining the ability to cause substantial damage^18^. These observations indicate that the capacity of microorganisms to adapt their metabolism in the context of BFI may support the maintenance of their pathogenicity. This notion is supported by an elegant model comparing 16 different bacterial–fungal pairs revealing new insights into the rewiring of metabolism and microbial fitness in BFIs^19^. In another study, integrated metabolomics and transcriptomics revealed antagonistic interactions between *Lactobacillus rhammosus* and *Candida albicans* (*C. albicans*), showing that bacterial overgrowth drives alterations of the metabolism of *C. albicans*, thus affecting fungal pathogenicity in intestinal epithelial cells^20^.

Here, we provide insights about metabolic networks operating during the physical interactions between *K. pneumoniae* and *A. fumigatus*. Using integrated multi-omics datasets, we uncover strategies and possible defense strategies that contribute to maintaining fitness and metabolism in *A. fumigatus* facing hostile environments. Our data reveal significant metabolic adaptations in *A. fumigatus* upon interaction with *K. pneumoniae*, highlighting regulators that influence the temporal dynamics of interspecies BFI. These data also reveal potential biomarkers that could be exploited to improve the clinical management of BFI.

## Results

### *A. fumigatus* activates catabolism to compensate for nutrient limitation during the interaction with *K. pneumoniae*

**Fig. 1:**
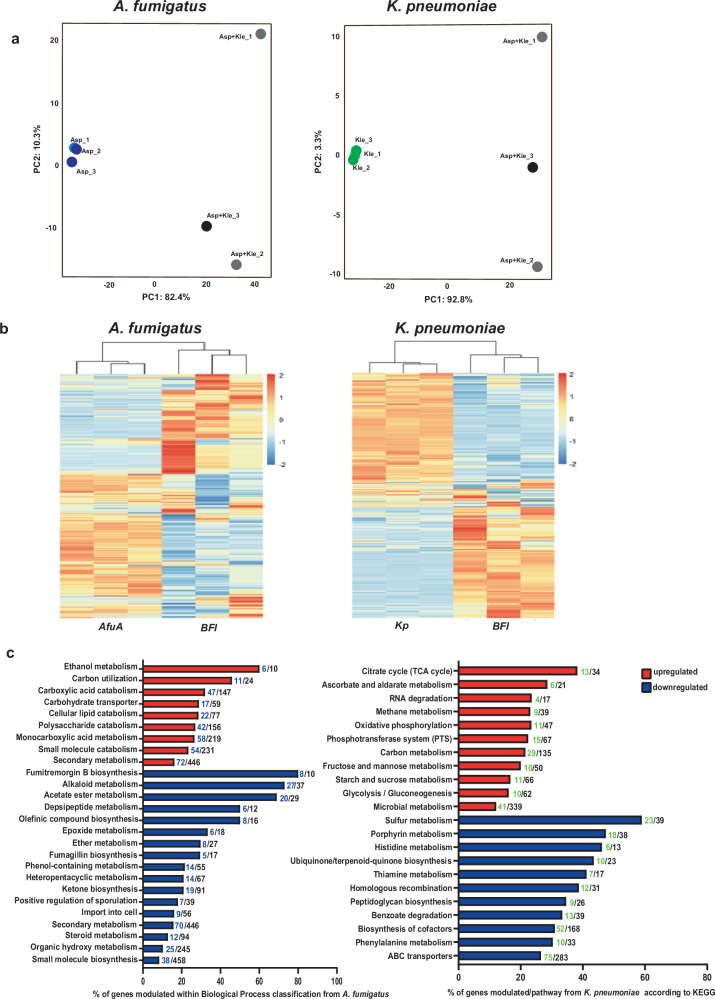
Transcriptomic profiling shows that overgrowth of *K. pneumoniae* triggers the activation of catabolic processes and alters carbon routes in *A. fumigatus.* **a** Principal component analysis (PCA) of transcripts obtained from BFI cultures and single pathogen cultures of *A. fumigatus* and *K. pneumoniae*. **b** Hierarchical clustering of differentially expressed genes (DEGs) of *A. fumigatus* and *K. pneumoniae*. The three biological replicates are shown for single pathogen cultures and co-cultures. The color code indicates the fold-change (log2) in gene expression. **c** Enrichment analysis of DEGs (log2 FC ± 1.5) showing the main biological processes modulated in response to BFI interaction in *A. fumigatus* (left side), according to Gene ontology (GO) terms and *K. pneumoniae* (right side) according to KEGG. Asp *A. fumigatus,* Kle *K. pneumoniae*. Numbers in front of the bars in blue or green indicate genes modulated in our dataset either in Afu or Kp, respectively, while numbers in black represent the total number of genes within that particular biological process.

To identify the interdependent responses during the BFI, we performed dual-RNA-seq of mixed bacterial–fungal biofilms. The transcriptomics data reflected an approximate minimum genome coverage of 12× and 111× for *A. fumigatus* and *K. pneumoniae*, respectively. The principal component analysis (PCA) verified the high quality of the biological replicates. Distinct clusters appeared for single and BFI biofilm samples. Notably, the BFI biofilm group exhibited greater variance when compared to the single biofilm group, which most likely is due to the increased complexity in BFI samples, such as distinct maturation stages present in the biofilm or altered responses in each microbial species. Further, time-dependent stochasticity mounting in BFI may further impact the variance, and thus modulate the number of regulated genes in a qualitative and quantitative manner. Nonetheless, the data showed clearly defined clusters specific to the BFI biofilm conditions (Fig. 1a). Hierarchical clustering based on normalized read counts emphasized distinct profiles between single pathogen and BFI cultures (Fig. 1b). The BFI compared to *A. fumigatus* alone showed 1642 differentially regulated genes (DEGs), with 1166 upregulated and 476 downregulated genes (Supplementary Fig. 1a). The BFI comparison with *K. pneumoniae* alone yielded 1561 DEGs, revealing 804 upregulated and 757 downregulated gene transcripts. The analysis highlighted the activation of catabolic routes of metabolism and ethanol metabolism in *A. fumigatus*, as well as a deregulation of biosynthetic metabolism and sporulation (Fig. 1c and Supplementary Fig. 1b). In *K*. *pneumoniae*, the central carbon metabolism was activated, with induction of glycolysis, TCA and oxidative phosphorylation, whereas pathways for amino acid synthesis, sulfur metabolism and cell wall construction were downregulated (Fig. 1c). The transcriptomics data imply dynamic metabolic alterations mounting in *A. fumigatus* to cope with the impact of overgrowing *K. pneumoniae* that promotes nutrient-starvation. These changes are emphasized mainly by the regulation of pathways that are required to utilize alternative carbon sources (Supplementary Table 1). Indeed, the top 20 DEGs in *A. fumigatus* pertain to carbon metabolism and secondary metabolism primarily related to gliotoxin biosynthesis and oxidoreductase genes (Supplementary Table 2). In contrast, the biosynthesis of alkaloid/secondary metabolism was downregulated (Supplementary Table 2). Regarding *K. pneumoniae*, most of the induced genes reflected central carbon metabolism and the translation machinery, while expression of the membrane transport system was diminished (Supplementary Table 3).

### Proteomics indicates translation alterations in *A. fumigatus* as an adaptive strategy upon BFI

**Fig. 2:**
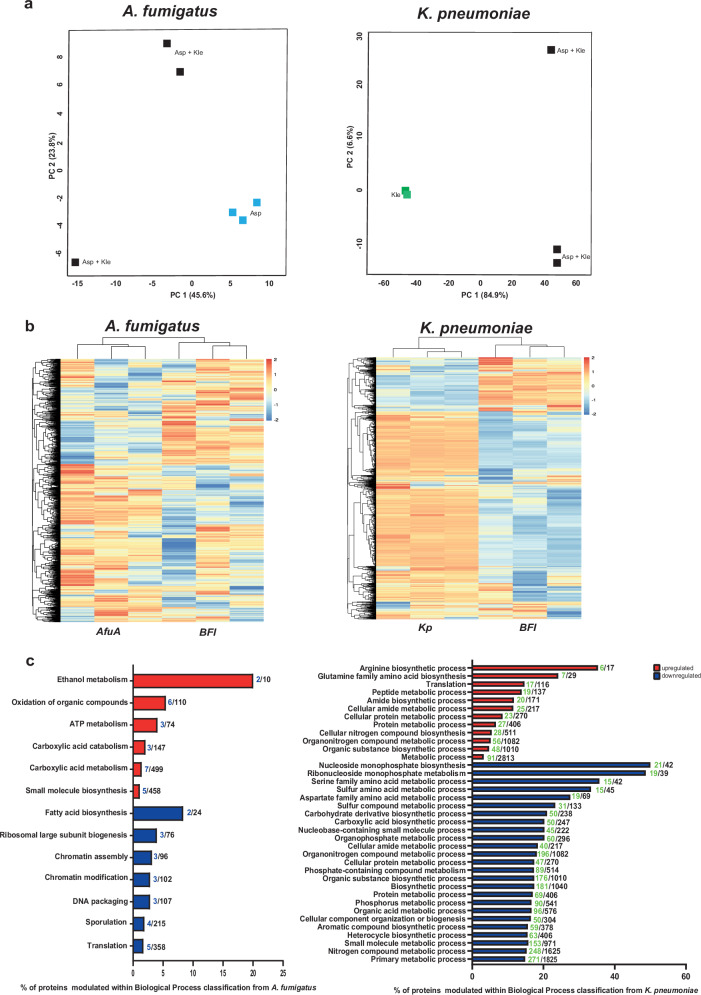
Proteomics data indicate that the bacterial–fungal interaction leads to changes in carbon metabolism in *A. fumigatus*, and fosters translation and amino acid metabolism in *K. pneumoniae*. **a** Principal component analysis of the proteome produced in BFI compared to each single pathogen culture. **b** Hierarchical clustering of differentially abundant proteins (DAPs) of *A. fumigatus* and *K. pneumoniae*. The three biological replicates are shown for single pathogen cultures and co-cultures. The color code indicates the fold-change (log2) in protein levels. **c** Functional enrichment analysis related to biological processes according to Gene ontology (GO) terms of the DAPs under BFI conditions compared to *A. fumigatus* (left) or *K. pneumoniae* (right). Asp *A. fumigatus*, Kle *K. pneumoniae*. Numbers in front of the bars in blue or green indicate proteins modulated in our dataset either in Afu or Kp, respectively, while numbers in black represent the total number of proteins within that particular biological process.

The proteomics data corroborated with the interdependent responses during the BFI showed a PCA with clear separation of samples from single pathogen vs BFI cultures (Fig. 2a), which was also confirmed by hierarchical clustering (Fig. 2b). Although the proteomics coverage was only about 30% of the total proteomes from both *K. pneumoniae* and *A. fumigatus* (Supplementary Fig. 2a), the data displayed 84 differentially abundant proteins (DAPs), as shown in (Supplementary Fig. 2b), and 1059 DAPs when comparing BFIs with single culture of *A. fumigatus* or *K. pneumoniae*, respectively.

A number of parameters may explain the low number of DAPs displayed by *A. fumigatus* upon the interaction with bacteria. For instance, a reduction of ribosome biogenesis that ultimately regulates the components of the translational machinery and translation. Furthermore, translation efficiency, co- and post-translational modifications, intracellular transport and stability, complex formation, may affect protein abundance in BFI. Notably, adaptive ribosome biogenesis is critical for proteostasis regulation in cells adapting to stress or nutrient limitations to compensate for the need of high energy costs in cells facing stress conditions^21^. Indeed, the functional enrichment of DAPs in *A. fumigatus* under BFI condition reveals a similar profile of deregulation of the translation machinery, sporulation, fatty acid synthesis and chromatin organization, reinforcing a shutdown of different energy-consuming processes (Fig. 2c and Supplementary Fig. 2c). Of note, alternative metabolic processes such as ethanol metabolism and carboxylic acid catabolism were induced (Fig. 2c and Supplementary Fig. 2c). Interestingly, *K. pneumoniae* sustained its central metabolism, and further activated peptide and amino acid biosynthesis pathways upon interaction with Afu (Fig. 2c). At the same time, sulfur-related metabolism, carboxylate metabolism and the biosynthesis of small compounds and aromatic compounds declined (Fig. 2c).

Hence, BFI conditions affected *A. fumigatus* in its energy metabolism and alternative carbon source utilization, including the upregulation of metabolic proteins such as Grg1, alcohol dehydrogenase, acetate kinase, malate dehydrogenase, succinate dehydrogenase, phosphatidyl synthase and pyruvate decarboxylase. Furthermore, BFI impacted fungal cell division and chromatin remodeling, including the downregulation of proteins such as sister chromatid cohesion/DNA repair (e.g., BimD) and nuclear localization (e.g., NPL6), rRNA processing (e.g., nucleolar protein 12), and ascospore formation (e.g., VPS13) (Supplementary Table S4). Proteins of higher abundance in *K. pneumoniae* were primarily related to translation, such as ribosomal proteins, carbon and nitrogen utilization, including glutamate and aspartate, lysine and ornithine transporters, as well as nitrogen regulatory proteins. Among downregulated bacterial proteins, the majority were involved in carboxylic acid metabolism, including acetolactate synthase, malate oxidoreductase and oxoglutarate (Supplementary Table S5).

### Metabolomics verifies adaptive routes in *A. fumigatus* in BFI and reveals candidate biomarkers

**Fig. 3:**
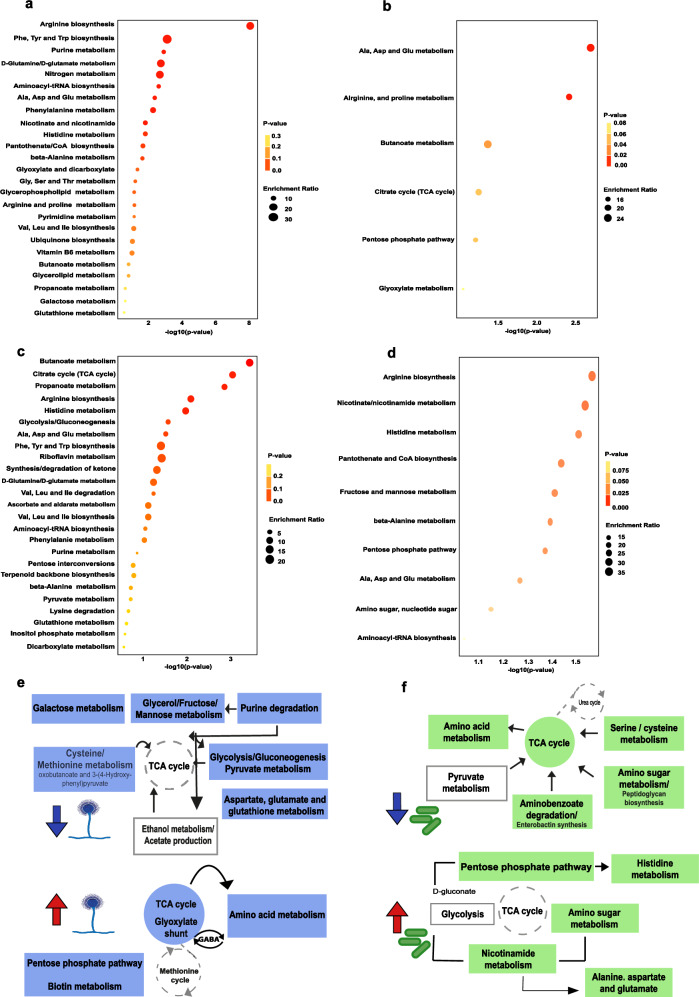
Metabolic routes adopted by *A. fumigatus* and *K. pneumoniae* upon bacterial–fungal interaction. Enrichment analysis of differentially regulated metabolites collected in supernatants from co-cultures after 24 h compared to the single pathogen culture of *A. fumigatus*: downregulated (**a**) and upregulated (**b**). Enrichment analysis of differentially regulated metabolites from co-cultures compared to single pathogen culture of *K. pneumoniae*: downregulated (**c**) and upregulated (**d**). Schematic representation of pathways potentially involved in *A. fumigatus* (**e**) and *K. pneumoniae* (**f**) adaptation under BFI stimuli. Blue and red arrows indicate down and upregulated general pathways, respectively. Gray boxes represent potential metabolic processes activated by produced metabolites. Dashed gray circles show possible metabolic processes involved in the entwined pathways. The enrichment analysis of paths responding to BFI was performed in MetaboAnalyst 5.0, using the KEGG database for the annotated metabolites with a log2 FC ± 1.0 cutoff.

The untargeted metabolomics approach was carried out to obtain an overview of the metabolism crosstalk during the BFI. In BFI supernatants, we identified 204 differentially modulated metabolites when compared to *A. fumigatus* single culture after 24 h. In *K. pneumoniae*, 256 metabolites were subject to changes during BFI. Depleted metabolites amounted to 126 and 227 for *A. fumigatus* and *K. pneumoniae*, respectively. These metabolites reflected specific pathways associated with fungal and bacterial adaptation to the environment emerging during BFI (Fig. 3 and Supplementary Fig. 3).

The functional categorization of Afu-depleted metabolites revealed a shift from glucose metabolism toward alternative sugar sources such as fructose, galactose and mannose. This shift is driven by a shortage of glucose, redirecting energy flow into different pathways, including concurrent involvement of glycerol metabolism, purine catabolism and uptake of amino acids. These adjustments secure nitrogen and carbon sources to meet the metabolic demands of the fungus (Fig. 3a and Supplementary Fig. 3a, b). Conversely, the 78 Afu-upregulated metabolites identified were mainly associated with ROS response, peroxisomal and mitochondrial activities (Fig. 3b and Supplementary Fig. 3c, d).

In *K. pneumoniae*, the enrichment analysis revealed a depletion of metabolites belonging to central energy pathways, such as glycolysis, pyruvate metabolism and the TCA cycle. We also observed a set of depleted metabolites related to amino acid, peptidoglycan and enterobactin biosynthesis (Fig. 3c and Supplementary Fig. 3e, f) indicating that the bacterium maintained favorable energetic routes. Although 29 bacterial metabolites found to be upregulated were indirectly linked to central energy routes revealing by-products of TCA and oxidative phosphorylation, processes of amino acid metabolism were predominant (Fig. 3d and Supplementary Fig 3e, f). An overview of the metabolic rearrangements in *A. fumigatus* and *K. pneumoniae* is shown in Fig. 3e and f, respectively. The most prominent metabolites produced by both pathogens upon BFI, revealing potential biomarker candidates, are listed in Supplementary Table 6.

Considering the set of upregulated metabolites in *A. fumigatus*, some compounds are potential candidate biomarkers for BFI, including ascorbic acid, a norfenefrine-mimic, hydroxycaproic acid, deoxyribose 5-phosphate, isopropylmalic acid and nipecotic acid. For *K. pneumoniae*, potential biomarker metabolites encompassed 6-methylnicotinamide, *α*-d-mannose 1-phosphate, l-aspartic acid, methamphetamine, amphetamine as well as gluconic acid.

### Secondary and alternative metabolism of *A. fumigatus* cooperate to maintain fungal fitness

**Fig. 4:**
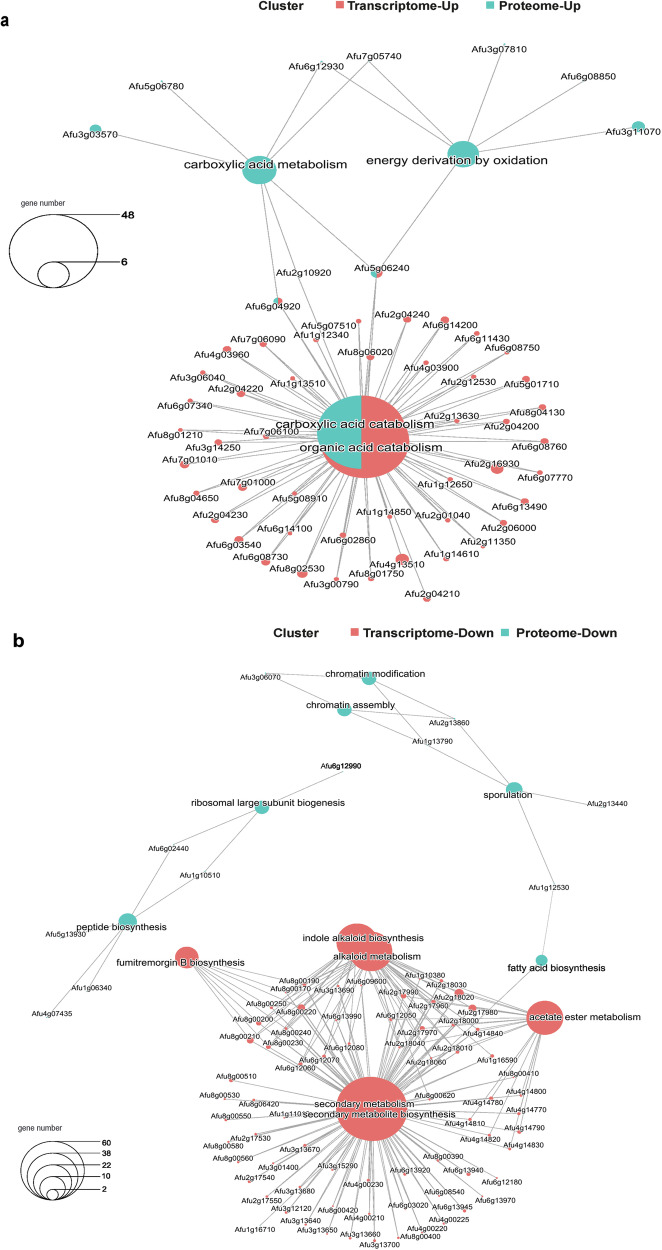
*A. fumigatus* adapts to the interaction with *K. pneumoniae* by deregulating the synthesis machinery and activating the bypass of energy routes. The interaction network of common processes upregulated (**a**) and downregulated (**b**) in *A. fumigatus*, with overlapping transcriptome (red) and proteome (blue) data, is shown.

To cope with the limited glucose supply, *A. fumigatus* repressed central carbon routes such as glycolysis, while catabolism was enhanced, as shown by the integration of proteomics and transcriptomics datasets (Fig. 4, Supplementary Fig. 4 and Supplementary Table 7). Among the markedly induced genes are those belonging to the beta-oxidation pathway, glyoxylate shunt and gluconeogenesis, such as enoyl-CoA hydratase, fatty-acyl coenzyme A oxidase (*pox1*), acyl-CoA dehydrogenase, 3-ketoacyl-CoA ketothiolase (*kat1*), isocitrate lyase (*icl*), malate synthase (*acuE*), phosphoenolpyruvate carboxykinase (*pck1*), fructose-1,6-bisphosphatase (*fbp1*) as well as the acetyl-CoA transporter genes (Supplementary Table 1). It is important to note that only a few genes and their corresponding protein products, including 3-oxoacyl-acyl-carrier protein, malate synthase and fatty acyl CoA oxidase, showed a divergence in expression levels across datasets (Table S1). This emphasizes temporal and spatial differences in the regulation and production of transcripts or proteins seen in *A. fumigatus*. By contrast, corresponding transcript and protein levels, including succinate dehydrogenase, aconitase hydratase or alcohol dehydrogenase showed high abundance, especially under emerging BFI conditions.

**Fig. 5:**
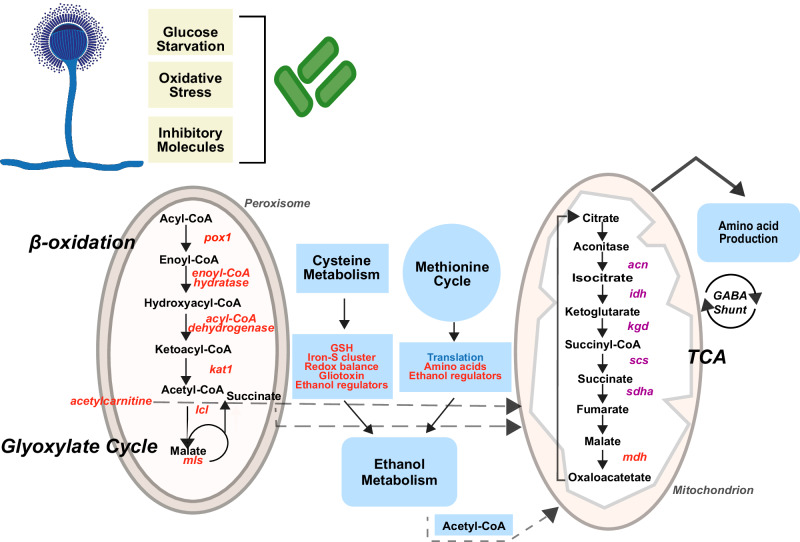
Schematic overview of the main strategies employed by *A. fumigatus* in response to the interaction with *K. pneumoniae.* Glucose starvation and inhibitory molecule production due to overgrowth of *K. pneumoniae* activates the catabolism of fatty acids as a source of acetyl-CoA supply. As a consequence, beta-oxidation and the glyoxylate cycle are induced, feeding the TCA cycle and keeping it activated. In parallel, the GABA shunt is also activated. The amino acid sources in the milieu are taken up by fungal cells and are transferred into the methionine and cysteine metabolism. The by-products can regulate ethanol production, which also supplies acetyl-CoA units to the TCA cycle. Methionine and cysteine metabolism can control amino acid production, regulate translation kinetics, enhance oxidative protection and boost gliotoxin production. Red letters indicate induced genes/proteins, products and processes, while purple letters reflect low-level induction. The translation deregulated process is indicated in blue letters. Dashed arrows reflect acetyl-CoA transport between cellular compartments.

**Fig. 6:**
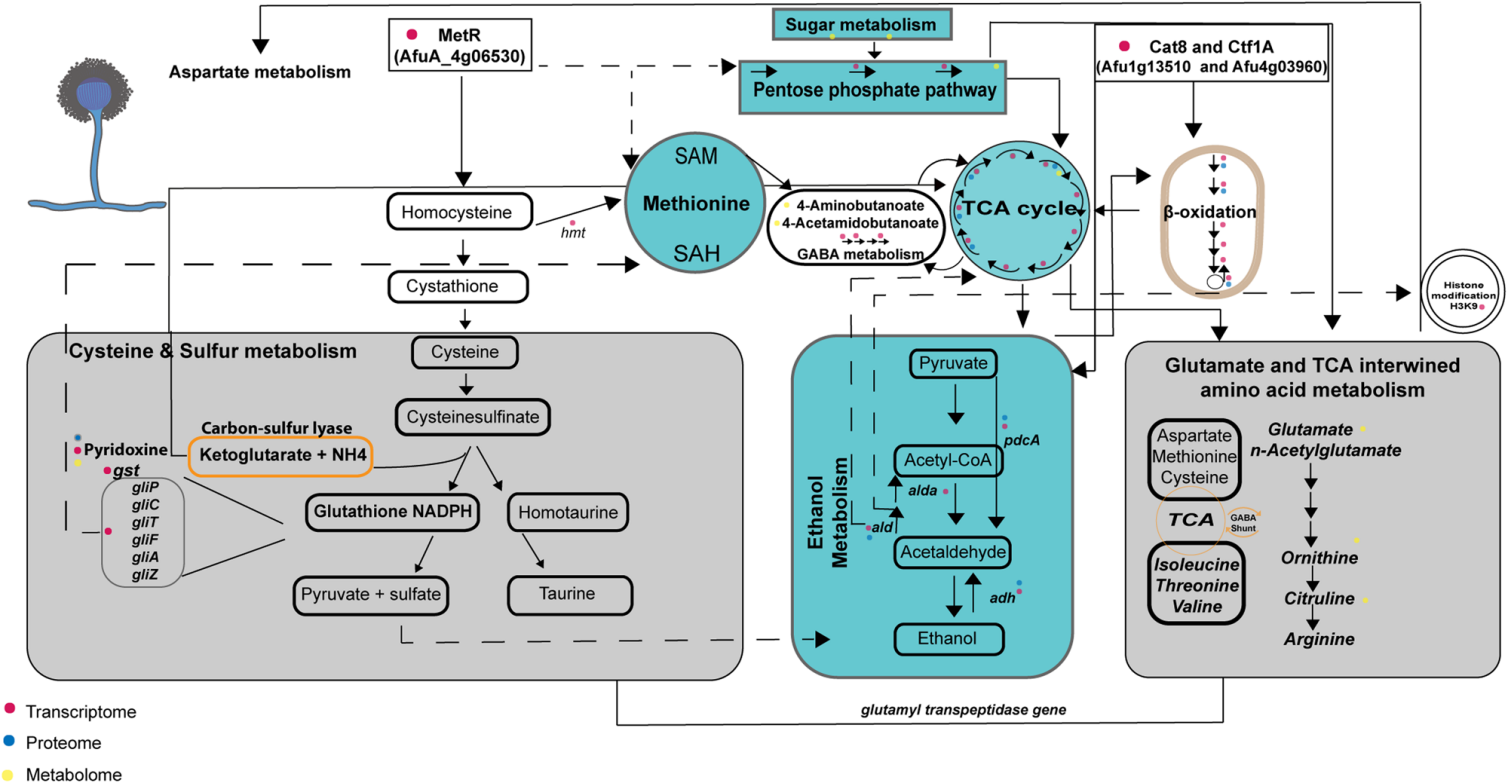
Schematic overview of adjustments in the metabolism of *A. fumigatus* upon interaction with *K. pneumoniae.* Our data suggest that MetR might act upstream of sulfur metabolism, PPP and methionine cycle by regulating these pathways directly or indirectly. Further adjustments are achieved by the connection between TCA, GABA metabolism and beta-oxidation. It is highly conceivable that gliotoxin-related products are associated with feedback regulation of the methionine cycle (indicated by black dashed arrows). Similarly, some carbon–sulfur compounds might regulate the TCA cycle (indicated by black arrow). The transcriptional regulators Cat8 and Ctf1 are shown to regulate the beta-oxidation cycle, which in turn can feed the TCA cycle (solid black arrows). TCA and GABA metabolism work together to provide energy sources and to regulate key amino acid production. The PPP pathway is also associated with the TCA cycle and amino acids biosynthesis (solid black arrow). Ethanol by-products might influence beta-oxidation and histone acetylation, which might in turn impact on translation efficiency (indicated by black dashed arrow). Orange rectangles or orange circles reveal possible sources for amino acid metabolism in *A. fumigatus*. Steps modulated within each pathway are indicated by red, blue and yellow dots according to their modulation revealed by transcriptomics, proteomics and metabolomics, respectively. Black dashed arrows indicate putative regulation or influence of one pathway or by-products on another pathway or a putative transcription factor regulation of paths that might cross. Direct regulation is shown by solid black arrows. *hmt* homocysteine *S*-methyltransferase, *pdcA* pyruvate decarboxylase, *ald* aldehyde dehydrogenase, *adh* alcohol dehydrogenase, *gst* glutathione *S*-transferase, *gliP* non-ribosomal peptide synthetase, *gliC* cytochrome P450 monooxygenase, *gliT* gliotoxin sulfhydryl oxidase, *gliF* cytochrome P450 monooxygenase, *gliA* glioxin transporter, *gliZ* Zn2Cys6 binuclear transcription factor.

It is noteworthy that the data revealed the upregulation of key players of fatty acid catabolism, including the transcription factor Cat8 (AfuA_1g13510) and Ctf1A (AfuA_4g03960) (Supplementary Table 1). Conversely, cell division, translation, spore formation and secondary metabolism primarily associated with conidia were downregulated (Fig. 4 and Supplementary Table 8). The shutdown of these pathways was a consequence of the metabolic shift, to save energy for the maintenance of cellular homeostasis allowing fungal adaptation. Remarkably, the data analysis indicated a connection of “secondary metabolism”, related to cysteine and sulfur metabolism, with primary metabolism involving methionine synthesis, the pentose phosphate pathway (PPP), ethanol metabolism, TCA, GABA metabolism and beta-oxidation (Figs. 5 and 6). The MetR transcription factor (AfuA_4g06530) possibly serves as an upstream control for sulfur metabolism, methionine cycle and PPP. The resulting metabolic products such as ketoglutarate, pyruvate and glutamate appear to feed into or regulate the TCA cycle, ethanol metabolism and amino acid biosynthesis (Fig. 6). Moreover, owing to the altered ethanol metabolism and acetate production, active beta-oxidation was increased. Adaptive chromatin changes possibly also played a role due to the effect of histone modification on transcription by altering translation efficiency through lysine acetylation (Fig. 6).

Regarding sulfur metabolism, downstream components such as the carbon–sulfur lyase genes, Fe–S cluster genes and gliotoxin-cluster genes were upregulated in *A. fumigatus* upon interaction with *K. pneumoniae* (Figs. 5, 6 and Supplementary Table 9). Antioxidant defense mechanisms were induced in *A. fumigatus* during BFI, including catalase (AfuA_8g01670) and superoxide dismutase *sod1* (AfuA_5g09240) (Supplementary Fig. 5). Our data revealed key transcription regulators (Cat8, Ctf1A and MetR) as crucial elements engaged in adaptation of *A. fumigatus* to the interaction with *K. pneumoniae*, as shown in Fig. 6 and in the validated dataset displayed in Supplementary Fig. 5.

## Discussion

The human body hosts trillions of microbial species that coexist within the tissue microbiota and are typically organized in heterogeneous mixed biofilms^22–25^. Tissue microbiota must offer conditions that enable fitness and access to nutrients to allow for physiological and homeostatic growth. Dysbiosis may arise from pathological interactions of microbial species but also from acute or chronic inflammatory host immune responses^26^. Hence, changes in the host immune status or in the availability of nutrients and microbial growth imbalances are often associated with disease^27,28^. For instance, inflammatory bowel diseases (IBD), which are chronic inflammatory disorders, are characterized by a breakdown of immune tolerance to the gut flora and an excessive immune response. This is mediated by different factors including the enhancement of pro-inflammatory bacterial communities in this particular niche^29,30^.

While complex polymicrobial infections have gained increasing attention, little is known about the molecular mechanisms, metabolic activities and interaction dynamics of this multi-microbial network. The integration of distinct omics datasets for probing the environment of polymicrobial interactions has provided powerful tools, enabling a better understanding of genetic regulatory networks and pathways operating in interactions between different species. This investigational strategy is expected to provide a platform paving the path to improved diagnostic and therapeutic approaches^31,32^. In our previous work^16^, we focused on molecular dissection of the interaction between *Aspergillus* spp. and *K. pneumoniae*, showing that the growth inhibitory effect of *K. pneumoniae* was mediated by blocking hyphal development. The inhibitory effect required physical contact with metabolically active bacteria^16^, although it cannot be excluded that soluble metabolites can contribute or even control the response^33–35^. Nonetheless, many molecular aspects underlying BFIs still need to be understood. Of note, competitive antagonistic or synergistic microbial interactions may display adverse clinical effects for the host^36–38^.

Here, we use integrated multi-omics data to identify metabolic responses and pathways operating during *A. fumigatus* and *K. pneumoniae* interaction. We identify fungal strategies that counteract potential inhibitory actions mounted by bacteria in BFI. The fungal adaptations not only ensure fitness and regulate growth, but also include metabolic and translational changes as integral requirements of survival and resistance within hostile surroundings such as BFI. Hence, the hallmarks in *A. fumigatus* responses that establish “survival” modes include a selective translation mode, environmental sensing and rewiring of the metabolism, as well as the connection of primary with “secondary” metabolism to achieve a “resiliency” when growing under BFI conditions.

The combination of omics datasets displayed a state of selective translation characterized by decreased biosynthesis, ribosome biogenesis and sporulation processes. These alterations presumably steer the fungal response to challenges posed by bacterial overgrowth. Indeed, it is feasible that adaptation to hostile environments and some defense factors trigger alterations in germination and translation processes, as shown previously^39^. For instance, during the interaction with macrophages at early time points, *C. albicans* exhibits decreased expression levels of genes associated with the translation machinery, peptide biosynthesis and translation regulators^40^. Moreover, interactions between *Aspergillus* spp. and *K. pneumoniae* displayed an impairment in fungal spore germination and hyphal formation followed by downregulation of the transcription factor calcineurin-responsive zinc finger and protein kinase A regulatory subunit (PKAR) encoding genes^16^ responsible for regulating conidial germination, hyphal growth and virulence^41,42^. Similarly, our data revealed a marked downregulation (log2 FC = −3.44) in the C2H2 transcription factor BrlA (AfuA_1g16590), which regulates the formation of conidia.

Further, an elegant recent report deciphered the interaction of the lung pathogens *Cryptococcus neoformans* (*C. neoformans*) with *K. pneumoniae* in co-culture with macrophages, demonstrating that *K. pneumoniae* cell numbers increase, whereas *C. neoformans* loads decrease. This suggests that the bacterium can fully maintain active metabolism and cell division, whereas the fungus must sense the emerging stress environment but requires more time to cope, perhaps owing to the longer generation time. Although the fungus adjusts translation, it can adapt by regulating processes related to catabolism, cellular regulation, translation and signaling^43^. Likewise, our data showed the bacteria strive to maintain an active metabolism by keeping the main routes ensuring their energy needs, including the TCA cycle, engaged. While these routes are important for providing by-products to meet cellular demands, these by-products also lead to redox-active compound production. In response to this milieu, *A. fumigatus* redirects its metabolism to satisfy additional energy requirements under the emerging stressful condition.

The main energy routes activated by *A. fumigatus* are beta-oxidation, glyoxylate and GABA shunts, gluconeogenesis, and ethanol metabolism (Table S1 and Figs. 5, 6). In the present analysis, the entire pathway of beta-oxidation was induced. The enzymes PCK and FBP1, representing rate-limiting steps in the glyoxylate shunt and the gluconeogenesis pathway, were also upregulated. Likewise, the GABA shunt pathway was activated reflecting circuit rearrangements to generate carbon and nitrogen sources, in this instance, through a bypass outside the classical TCA. Since the GABA shunt is energetically less favorable for the cells^44^, it is difficult to imagine its operation in the absence of stress conditions. However, in a BFI environment, it is conceivable that this operation occurred due to glucose depletion and the ensuing need for alternative energy sources to maintain cellular demands and to support fungal adaptation.

Moreover, key regulators responsible for reshaping Afu metabolism, such as Cat8/FacB, and Ctf1A, were upregulated in our analysis (Supplementary Table 1). These regulators act under the conditions of glucose depletion and allow for the de-repression of catabolite suppression by promoting alternative carbon source utilization. Moreover, the Cat8/FacB is responsible for ethanol and acetate utilization and, in consequence, feeding and regulating aspartate, glutamate, TCA and glyoxylate metabolic processes as well as secretion of secondary metabolites^45^.

Notably, ethanol metabolism is enhanced upon interaction with *K. pneumoniae*. It has been demonstrated that ethanol levels are increased in the lungs of murine models for invasive pulmonary Aspergillosis^46^. This substantiates the notion that ethanol serves as a potential carbon source for *A. fumigatus* during infection and other stressful conditions. Moreover, by-products of ethanol catabolites have an effect on bacterial partners by affecting growth, biofilm formation, virulence and drug resistance^35^.

Ultimately, changes in Afu metabolism and carbon routes can lead to changes in the cell wall composition, architecture and hydrophobicity. These potentially affect drug resistance, virulence, immune recognition and evasion^47–50^ and the fungal–bacterial interaction itself^51^. Indeed, BFI between *A. fumigatus* and *K. pneumoniae* has been shown to trigger changes in fungal cell wall genes, including *chs3*, *exg1* and *mpkC*^16^. These observations are corroborated by the present data revealing cell wall components such as chitinases and glucanases as DEGs (Supplementary Table 1).

In addition to changes in alternative carbon sources to meet energy needs, Afu also responds by activating stress-adapted metabolic processes, linking primary and “secondary” metabolism. As outlined before, bacterial metabolism induces the production of pro-oxidant compounds. To cope with the stressful milieu, PPP and sulfur pathways are activated, both offering pleiotropic functions, enabling *A. fumigatus* to maintain fitness and homeostasis. PPP has been shown to provide acetyl-CoA for the TCA cycle and respond to ROS stimuli^52^ and can link fatty acid utilization to sulfur metabolism^53^. Our data show that Afu upregulates 2-deoxy-d-ribose 5-phosphate metabolite, as well as phosphogluconate dehydrogenase (AfuA_6g08730, AfuA_5g01250, AfuA_5g10280), a putative 6-phosphogluconolactonase (AfuA_1g02980), and the transcription regulator MetR upon BFI (Supplementary Table 1). In this regard, our data imply that MetR is a potential regulator acting upstream of the methionine cycle, PPP, cysteine and sulfur pathways.

Regarding sulfur metabolism and sulfur-derived compounds, it has been widely accepted that they are crucial for the viability, virulence and iron homeostasis of *Aspergillus* spp.^54^, as exemplified by S-containing molecules such as vitamin thiamine or *S*-adenosylmethionine^55,56^. Of note, sulfur is also required for producing ergothioneine and glutathione, and it is involved in gliotoxin biogenesis^57^. Moreover, sulfur plays a role in iron homeostasis and sensing via the glutaredoxin GrxD^58^. Upon BFI, we show the upregulation of gliotoxin biogenesis and the protective antioxidant defenses, including the modulation of catalase and superoxide dismutase genes. In addition, our data reveal the upregulation of siderophore biosynthesis protein (AfuA_1g04450), involved in ferricrocin and hydroxyl-ferricrocin production. Both siderophores play a role in iron storage and transport through the hyphae or conidia in fungal cells^59^. The production of gliotoxin after *A. fumigatus* interaction with *K. pneumoniae* was recently reported^17^. Our data are consistent with previous studies showing that gliotoxin is not solely acting as a toxic and/or antioxidant molecule, it can also play a significant role in regulating primary metabolism by affecting methionine cycle^60^.

Finally, our data shed light on the plasticity of *A. fumigatus* metabolism when exposed to interaction with bacteria. Although, we are well aware about potential limitations that apply to the in vitro model and the media selection used to address the BFI, mainly due to the absence of human immune defense, the conditions chosen here, suggest extraordinary complexity and dynamics in BFI. Thus, we feel that adding additional factors might result in datasets that cannot be handled properly. Artificial intelligence approaches may be the way to go in future experiments, but we felt quite strongly that our initial setup should adhere as much as possible to standardized and reproducible in vitro conditions. Of course, the “optimal” medium to perform BFI experiments would be lung-mimicking conditioned media^61^ or exudates from patients suffering from co-infections. However, such approaches would have severe limitations and confounding effects owing to variations in the immune defense present in distinct human samples. The data presented reveal how metabolic networks are adapted by *A. fumigatus* during BFI, demonstrating the metabolic plasticity of pathogens in mixed biofilms that impose severe stress conditions and nutrient limitations. The observations also pose some important questions, requiring future efforts. For example, can highly adapted *A. fumigatus* better evade the host immune defense and thus become hypervirulent? Could the differences between pathogenic or stress-adapted metabolism of *A. fumigatus* explain discrepancies observed between BFI in vitro and in vivo? Importantly, our findings disclose possible targets amenable for clinical intervention to combat BFI. These include transcription factors that regulate hallmark responses as well as metabolite pathways emerging upon the interaction that might be exploited for diagnostic and ultimately therapeutic improvements.

## Methods

### Strains and growth conditions

*Aspergillus fumigatus* ATCC 204305 and *K. pneumoniae* ATCC 700603 are the strains used in this study. *A. fumigatus* was maintained in malt extract agar (MEA, SIGMA) plates for 3–4 days at 37 °C. Spores were collected into 1x PBS + 0.1% Tween 20, then filtrated by using a 40 μm cell strainer. Spores were stored at 4 °C for up to 1 week for subsequent use in the experiments. *K. pneumoniae* strains were maintained in Luria-Bertani (LB) agar plates and grown for 12–16 h in liquid LB medium at 37 °C with agitation at 180 rpm. The co-cultures of *A. fumigatus* and *K. pneumoniae* were carried out adhering to conditions reported elsewhere^16^. Basically, an equal cell numbers of *A. fumigatus* and *K. pneumoniae* (10^6^ cells/mL) were used in co-culture experiments. For proteomic, transcriptomic and metabolomic analyses, single pathogen cultures and co-cultures of *A. fumigatus* and *K. pneumoniae* were grown as biofilms, with static incubation in 35 × 10 mm tissue culture dishes (CytoOne, Starlab GmbH, Ahrensburg, Germany). *K. pneumoniae* was added after pre-germination of *A. fumigatus* spores, permitting the formation of hyphae for up to 12 h. Growth was allowed for 24 h at 37 °C in 3 mL medium. YNBP minimal medium (1.7 g/L yeast nitrogen base (YNB) without amino acids, 5 g/L ammonium sulfate, 25 mM phosphate buffer pH 7.0 (KH_2_PO_4_ + K_2_HPO_4_), 2.5 mM *N*-acetyl-glucosamine, 0.2% glucose, 0.1% maltose) was used for proteomic and metabolomic analyses, whereas sterile-filtered yeast extract peptone dextrose (YPD) medium (10 g/L yeast extract, 20 g/L peptone, 2% glucose) (Formedium, Norfolk, UK) was used for transcriptomic analysis. The rationale of selecting different growth media for transcriptomics analysis was to assure optimal growth conditions for each species and to recover RNA amounts that are sufficient and suitable for RNA-seq.

### Transcriptomics and bioinformatics workflow

Upon 24 h growth in biofilm mode, cells of single pathogen cultures and co-cultures of *A. fumigatus* and *K. pneumoniae* were collected in centrifuge tubes by refrigerated (4 °C) centrifugation at 3000 × *g*. Cells were then washed two times with 1x PBS and collected into 2 mL fast prep tubes with cold water. The supernatant was removed and cell pellets were used for RNA isolation. Pellets were resuspended in 1 mL Trizol (SIGMA) for 5 min at room temperature. A scoop of 0.3 g RNAse-free glass beads (SIGMA) was added to the sample and further incubation for 5 min at room temperature was performed. Following mechanical disruption by FastPrep for 2 × cycles 6.0 m/s, 45 s, samples were spun down at 4 °C for 15 min using 16,912 × *g*. The upper phase was transferred into new microtubes and 200 µL chloroform were added. The tubes were shaken vigorously by hand for 15 s with subsequent incubation for 3 min at room temperature. Samples were spun down at 16,912 × *g* for 15 min at 4 °C, and the aqueous phase was transferred into a fresh microtube. A volume of 500 µL of cold isopropanol was added and samples were incubated on ice for 20 min, followed by centrifugation at 16,912 × *g* for 30 min at 4 °C. The supernatant was discarded, and pellets were washed with 1 mL of cold 70% ethanol, and subsequently centrifuged at 16,912 × *g* for 5 min at 4 °C. Most of the ethanol was removed and another centrifugation step was performed to remove the remaining ethanol. Pellets were air-dried for 1 min and the resulting RNA was dissolved in 20 µL RNAse-free water (Invitrogen). The concentration of RNA samples was determined, and 15 µg were used for DNAse digestion. The enzyme DNAse I (Roche), the appropriate buffer and Ribolock RNAse inhibitor (Fermentas) were used. Samples were incubated for 30 min at 37 °C, and RNAse-free water was added to each sample. Purification of 100 µL RNA was performed by using the RNeasy kit (Qiagen) according to the respective manufacturer instructions. The quality of the RNA samples was checked on a Bioanalyzer using RNA 6000 Nanochips (Agilent). A control PCR assay was performed to check for the absence of residual DNA in the samples. Thereafter, RNA samples were processed for cDNA synthesis using 1 µg RNA by the RevertAid Reverse Transcriptase (Fermentas). Prior to RNA sequencing, an mRNA library was prepared by using a RiboZero epidemiology kit (NEB Ultra) for rRNA depletion. Sequencing was performed in 50 bp single-end mode on a HiSeq 4000 instrument at the NGS VBC Facility.

For annotation purposes, the strain *Klebsiella pneumoniae* subs*. pneumoniae* MGH 78578 (https://www.genome.jp/kegg-bin/show_organism?org=T00566) was used, as it is better annotated than the experimentally used strain *Klebsiella quasipneumoniae* (*K. quasipneumoniae*) ATCC 700603 (https://www.genome.jp/kegg-bin/show_organism?org=T04389). Additional information on annotations was also retrieved from UniProt. Prior to the analysis of the co-cultures, the genomes of *A. fumigatus* and *K. pneumoniae* were retrieved from NCBI and merged. For this, *Aspergillus fumigatus* Af293, genome version s03-m05-r06 and *K. quasipneumoniae* ATCC 700603, NCBI_Assembly GCF_001596075.2, sequence-region NZ_CP014696.2 1 5284734 were used. Quality control (QC) of raw sequencing reads was done using fastQC v0.11.8 160 (http://www.bioinformatics.babraham.ac.uk/projects/fastqc/). TrueSeq (Illumina) adapters were trimmed using the Cutadapt v1.18 tool (https://cutadapt.readthedocs.io/en/stable/). Reads were mapped onto genomes of the merged strain Af293 s03-m05-r06 and ATCC 700603 GCF_001596075.2 genomes using NextGenMap v0.4.12^62^. Reads mapping to rRNA *loci* were removed. Read counts were determined with HTSeq count v0.6.1p1 using the union mode^63^ and reference annotations for Af293s03-m05-r06 ATCC 700603 GCF_001596075.2, respectively. Pairwise comparison of differential expression analysis of single pathogen cultures with co-cultures was employed using edgeR v3.40.2 using R v4.2.2^64^. Benjamini–Hochberg adjusted *p* values were used to determine DEGs^65^. PCA using normalized read counts (CPM) was done using the R stats package v3.4.4. Hierarchical clustering was performed using the “pheatmap” package in R^66^. Gene ontology (GO) analysis was performed by using KEGG Mapper for *K. pneumoniae* and AspGD (*Aspergillus* genome database) GO Slim Process Mapper and FungiFun, GO classification ontology in *A. fumigatus*. Summarization of GO terms was carried out using REVIGO^67^. Gene set enrichment analysis was performed with the “clusterProfiler” package in R.

### Proteomics

Upon 24 h growth in biofilm mode, pellets of single pathogen cultures and co-cultures of *A. fumigatus* and *K. pneumoniae* were collected and washed with 1x PBS. The protein extraction was based on mechanical disruption with lysis buffer followed by TCA precipitation. Briefly, two volumes of ice-cold lysis buffer [8 M urea, 20 mM Tris, 20 mM DTT] and a scoop of glass beads were added to the samples, with ensuing fast prep for two cycles at 6.0 m/s for 45 s. After centrifugation for 10 min at 16,912 × *g* at 4 °C, the upper phase was transferred into new microtubes. The TCA precipitation was based on Cold Spring Harbor Protocols^46^, with some modifications. A mix of 13.3% TCA (w/v) (Merck), 0.093% 2-mercaptoethanol (v/v) and acetone (ice-cold) was added to the samples and overnight incubation at 4 °C was performed. Precipitates were recovered by centrifugation using maximum speed (25,000 × *g*) for 15 min at 4 °C, and supernatants were discarded. Pellets were washed in 0.07% 2-mercaptoethanol (v/v) + 80% acetone (ice-cold) followed by centrifugation at maximum speed (25,000 × *g*) for 10 min at 4 °C, and supernatants were discarded. This step was performed twice. Finally, protein pellets were air-dried at room temperature for 5 min, and frozen at −80 °C until use.

Frozen pellets were dissolved in 50 µL of 8 M urea/50 mM ammonium bicarbonate (ABC) solution. The protein concentration was estimated using a tryptophan fluorescence assay^68^. After reduction (10 mM dithiothreitol, 30 min at room temperature), alkylation (20 mM iodoacetamide, 30 min at room temperature in the dark), and quenching of excess iodoacetamide with 5 mM dithiothreitol (10 min at room temperature in the dark), the sample was diluted into 4 M urea with 50 mM ABC before adding 1 µg of endoproteinase Lys-C (Wako) for protein digestion. The digestion was performed at room temperature for 2 h, and the sample was further diluted into 1 M urea with 50 mM ABC, and 2 µg Trypsin (Trypsin Gold, Promega) were added. After overnight incubation at 37 °C, the samples were acidified with 1% trifluoroacetic acid (final concentration) and the peptide solution was desalted using a stage-tip protocol with Empore SPE C18^69^. Peptides were separated on an Ultimate 3000 RSLC nano-flow chromatography system (Thermo Fisher) using a pre-column for sample loading (Acclaim PepMap C18. 2 cm × 0.1 mm, 5 μm, Thermo Fisher), and a C18 analytical column (Acclaim PepMap C18.50 cm × 0.75 mm, 2 μm, Thermo Fisher), applying a segmented linear gradient from 2% to 80% solvent B (80% acetonitrile, 0.1% formic acid; solvent A 0.1% formic acid) at a flow rate of 230 nL/min for 120 min. Eluted peptides were analyzed on a Q-Exactive HF Orbitrap mass spectrometer (Thermo Fisher), which was coupled to the column with a nano-spray Flex ion-source (Thermo Fisher) using coated emitter tips (New Objective). The mass spectrometer was operated in a data-dependent mode, survey scans were obtained in a mass range of 380–1500 m/z, at a resolution of 120k at 200 m/z and an AGC target value of 3E6. The 20 most intense ions were selected with an isolation width of 1.6 Da, fragmented in the HCD cell at 27% normalized collision energy and the spectra recorded at a target value of 1E5 and a resolution of 15k. Peptides with a charge of +1 or >+6 were excluded from fragmentation, the peptide match feature was set to preferred, the exclude isotope feature was enabled, and selected precursors were dynamically excluded via repeated sampling for 40 s.

Acquired data were searched with MaxQuant 1.5.5.1^70^ against the UniProt reference databases for *A. fumigatus* (*Neosartorya fumigata*—strain ATCC MYA-4609) and *Klebsiella pneumoniae* (strain ATCC 700721/MGH 78578) and a custom database of common lab contaminants. We used the default parameters with full trypsin specificity, and with oxidation (M) and N-term acetylation as variable, and carbamidomethylation (C) as fixed modifications. Label-free quantification and “match-between-runs” functions were enabled, all data were filtered at 1% false discovery rate for peptide spectrum matches, proteins, and modified sites. Data were imported into Perseus for further analysis^71^. Version 1.5.0.15 was used for the analysis of *A. fumigatus* and the version 1.6.0.7 for the analysis of *K. pneumoniae*. Data of protein groups were log-transformed and filtered for at least two quantified data points per group before imputation of missing values (from normal distribution, with a downshift of 1.8 and a width of 0.3 standard deviations). As the coverage of *Klebsiella* proteins in the co-culture samples was much lower due to the lower abundance, the normalization procedure of MaxQuant was not sufficient. We therefore performed re-normalization by median-centering and then imputed missing values of *Klebsiella* proteins from a simulated normal distribution, as described above, but based only on the distribution of the *Klebsiella* protein group intensities. LIMMA statistical analysis was performed in R for non-imputed and imputed datasets, respectively. *p* values were adjusted for multiple testing with the Benjamini–Hochberg procedure in the LIMMA package.

Functional annotation was performed using imputed LFQ data, including only the significant proteins (LIMMA < 5% FDR). Bioinformatics Resources software^72,73^ was used for the protein functional annotation. The version 6.8 was used for *A. fumigatus* and the version 6.7 was used for *K. pneumoniae* analysis. The total number of proteins is related to the median protein counts for each reference genome, retrieved from NCBI on 10th April 2020. The functional categorization was performed according to GO terms and the Kegg pathway. The Revigo Web server (http://revigo.irb.hr/) was used for the summarization of GO terms^67^.

### Metabolomics

Upon 24 h growth in biofilm mode, 1 mL of medium was removed from each Petri dish, and the remaining supernatant plus the entire number of cells from single pathogen cultures and co-cultures of *A. fumigatus* and *K. pneumoniae* were collected into centrifuge tubes. To prepare for the cell pellet extraction, 9 mL of a cold solvent mixture [MeOH: ACN: H_2_O] (2:2:1, v/v) (methanol: acetonitrile: Milli-Q water) were added to the tubes. Samples were shaken vigorously for 30 s and incubated in liquid nitrogen for 1 min. Samples were then thawed at room temperature and sonicated in a water bath Bioruptor (Diagenode) at maximum power, 10 cycles, 30 s + 30 s rest in a water/Mili-Q water/ice bath. Incubation in liquid nitrogen and sonication were repeated three times. For protein precipitation, samples were incubated at −20 °C for 1 h. Thereafter, cells were shaken vigorously for 1 min and spun down at maximum speed (3000 × *g*) for 15 min at 4 °C. A volume of 1 mL supernatant was collected into microtubes and spun down for 15 min at 15,682 × *g* at 4 °C. The supernatants were transferred into fresh microtubes and evaporated by drying at room temperature in a vacuum concentrator. The dry extracts were pooled, reconstituted in 150 µL of [ACN: H_2_O] (1:1, v/v) and mixed with a pipette. Samples were sonicated in a water bath Bioruptor (Diagenode) at maximum power, 10 cycles, 30 s + 30 s rest in a water/Milli-Q water/ice bath. Samples were then spun down for 15 min at 15,682 × *g* at 4 °C to remove insoluble debris, and the supernatants were transferred into fresh microtubes. All samples were stored at −80 °C until further LC–MS analysis.

Extracted samples and the pooled QC were diluted 1:5 with 80% ACN for hydrophilic interaction liquid chromatography (HILIC) analysis and 1:5 with 50% ACN for reversed-phase (RP) analysis. Samples were separated on a SeQuant ZIC-pHILIC HPLC column (Merck, 5 µm, 100 × 2.1 mm) or a Phenomenex Gemini column (C18, 3 µm, 110 Å, 150 × 2 mm) with an UltiMate 3000 RS UHPLC system (Thermo Scientific, Bremen, Germany). The gradient ramp-up time for HILIC measurements was 20 min from 90% A (ACN) and 10% B (20 mM NH_4_HCO_3_) to 60% B and hold for 8 min. The total run time was 40 min at a flow rate of 100 µL/min. The starting mobile phase composition for RP measurements was 0.1% FA (A, 99%) and 0.1%FA in ACN (B, 1%). The ramp-up time was 20 min to 60% B and hold for 5 min. Total run time was 35 min at a flow rate of 100 µL/min. Metabolites were ionized by electrospray ionization in polarity switching mode after HILIC and in positive polarity after RP separation. Spectra were acquired by data-dependent high-resolution tandem mass spectrometry on a Q-Exactive Focus (Thermo Fisher Scientific, Germany). Ionization potential was set to +3.5/−3.0 kV, the sheet gas flow was set to 20, and an auxiliary gas flow of 5 was used.

Metabolite annotation was supported by the software Compound Discoverer 2.1 (Thermo Fisher Scientific). Compound annotation was conducted by searching the mzCloud database with a mass accuracy of 3 ppm for precursor masses and 10 ppm for fragment ion masses as well as ChemSpider with a mass accuracy of 3 ppm searching KEGG, Human, *Escherichia coli*, or Yeast Metabolome Database. Data were exported into Excel files. Different LC–MS experiments were represented and divided according to the separation mechanism (HILIC, RP) and polarity (+/−, only applicable for HILIC). All metabolites were sorted according to their molecular weight. Metabolites that were annotated with mzCloud (MS^2^ match at least 60%) were exported to the Excel file without filter adjustment and with a visible name. Identification was based on comparison of measured MS^2^ spectra in mzCloud. Metabolites containing at least one ChemSpider identification (CSID)—through molecular weight matching (max. tolerance 3 ppm)—were listed separately after mzCloud compounds and were filtered as follows: (i) group coefficient variant (Group CV) not exceeding 25% for −HILIC and 50% for +HILIC and RP in at least one sample group, (ii) log2-fold change either less than −1 or greater than +1 in at least one sample group, and (iii) further information on a specific compound was retrieved from ChemSpider online (http://www.chemspider.com/) using the CSID numbers. Annotation was performed by mzCloud or ChemSpider, depending on the indication of a name, CS, “CS or Similar to:”, and if “Similar to:” was indicated, mzCloud found a similar structure. If the name field remained empty, neither mzCloud nor ChemSpider could identify the molecular weight. There was no similarity search in −HILIC. Some metabolites were annotated identically, but had a different retention time, and could not be distinguished by the evaluation program. They most likely represented isomers or in-source fragments from other metabolites.

Differences in the relative amounts of metabolites produced at significant levels in co-culture were compared to the corresponding single pathogen culture. The group areas corresponding to single pathogen cultures and co-cultures were determined by Compound Discoverer. The non-quantitative nature of metabolomic analysis precluded a normalization strategy. An adjustment of the data values was therefore performed, considering technical differences between the biological replicates and between the samples (single pathogen cultures vs co-cultures). The maximum value for the coefficient of variation (CV) was 164%, corresponding to the maximum dispersion of data across all samples, and reflecting the standard deviation of the mean. To ensure that only relevant metabolites are considered, a cutoff for the log2-fold change (FC) > 1.0 was applied. Initially, the measured peak areas for single cultures of *Klebsiella* and *Aspergillus* were compared to determine which pathogen is the potential producer of a certain metabolite, and the values were subsequently compared with values measured in the co-cultures. Molecules displaying a specific monoisotopic mass were annotated according to their function by several online tools including PubChem (https://pubchem.ncbi.nlm.nih.gov/), ChemSpider (http://www.chemspider.com/), KEGG, BioCyc (https://biocyc.org/). The functional categorization was performed for annotated compounds in MetaboAnalyst 5.0^74^ across KEGG pathways. The hypergeometric test was used for pathways analysis. An overview of the metabolic analysis is provided in Supplementary Data 1.

### Primer design and qPCR

For gene expression analysis by qPCR, target sequences of *A. fumigatus* were retrieved from the *Aspergillus* genome database (http://www.aspgd.org/ or https://fungi.ensembl.org/Aspergillus_fumigatus). Primer design was performed by using the software PerlPrimer (version 1.1.21) (Open-source PCR primer design, Parkville, Australia)^75^ and IDT DNA primer quest tool (www.idtdna.com/primerquest/Home/Index), and the sequences are listed in Supplementary Table 10. The qPCR experiments were performed with SYBR green master mix (Applied Biosystems) in the Bio-Rad CFX Maestro platform. The algorithm used for gene expression analysis was the relative quantification ∆∆CT method^76^, using β-tubulin^77^ as the normalizer and *A. fumigatus* single culture as the reference of modulation. The graphs were generated using GraphPad Prism v.5 software (GraphPad). The results are displayed as mean values from independent experiments and the standard deviations. Significant values were determined using unpaired *t*-test. *P* values < 0.05 were considered significant.

### Statistics and reproducibility

DEGs were determined using edgeR v3.40.2 in R with Benjamini–Hochberg adjusted *p* values. Normalization of proteomics data was performed using MaxQuant 1.5.5.1 and the LIMMA package in R, with *p* values adjusted for multiple testing using the Benjamini–Hochberg procedure. For metabolomic analysis, due to its inherent non-quantitative nature, normalization was not applied. However, data values were adjusted to account for technical differences between the three biological replicates and between samples (single pathogen cultures vs co-cultures). The maximum CV was 164%, reflecting the maximum dispersion of data across all samples, representing the standard deviation from the mean. A hypergeometric test was used for pathway analysis in MetaboAnalyst 5.0. For qPCR validation, we used the relative quantification ∆∆CT method normalized to β-tubulin and *A. fumigatus* single culture as reference for changes. Graphs were generated using GraphPad Prism v5 software. The results are displayed as mean values from independent experiments, with standard deviations. Significant differences were determined using an unpaired *t*-test with *p* values < 0.05 as significant.

## Supplementary information

Supplementary Material

Description of Additional Supplementary Files

Supplementary Data 1

Supplementary Data 2

## Data Availability

The datasets can be found in online repositories. The RNA-seq data are available at the Gene Expression Omnibus (http://www.ncbi.nlm.nih.gov/geo) under the accession number: GSE199079. The proteomics data from this study are available at Pride database under the accession code (PXD046439). Source data are provided with this paper as Supplementary Data 2.

## Code availability

The code applied for dual-RNA-seq data analysis is available on GitHub and has been archived via Zenodo. It can be accessed through the following link: 10.5281/zenodo.13941729^78^.
