# Supplementary material for: Integrated multi-omics identifies pathways governing interspecies interaction between A. fumigatus and K. pneumoniae
Source: Commun Biol. 2024 Nov 12;7:1496. doi: 10.1038/s42003-024-07145-x (PMC11557599; doi:10.1038/s42003-024-07145-x)
Supplement: Supplementary file 1 — Supplementary Material [file 42003_2024_7145_MOESM1_ESM.pdf]

Integrated multi-omics identifies interaction pathways governing interspecies interaction between *A. fumigatus* and *K. pneumoniae*

Tamires Bitencourt [1,2,3§], Filomena Nogueira[1,2,3§], Sabrina Jenull [3,4§], Phan-Canh Trinh[3], Michael Tscherner [3], Karl Kuchler [3*] & Thomas Lion[1,2,5*]

From the

[1]CCRI – St. Anna Children's Cancer Research Institute, Vienna, Austria,

[2]Labdia - Labordiagnostik GmbH, Vienna, Austria,

[3]Medical University of Vienna, Max Perutz Labs Vienna, Department of Medical Biochemistry, Campus Vienna Biocenter, Vienna, Austria,

[4]Institute of Microbiology, Department of Pathobiology, University of Veterinary Medicine Vienna, Vienna, Austria,

[5]Department of Pediatrics, Medical University of Vienna, Vienna, Austria.

Supplementary information table of contents

Supplementary Figure 1. Page 4

Overview of the differentially expressed genes (DEGs) in *A. fumigatus* under bacterial-fungal interaction conditions and the enriched modulated processes according to GSEA enrichment analysis. a, Volcano plot representing the up- and downregulated DEGs is shown, using log2FC±1.5 cut off and p<0.05, upon BFI. **b**, GSEA analysis covering the biological process: carboxylic acid catabolism and ribosome biogenesis determined by transcriptomic analysis. Up- and downregulated genes are shown by red and blue colors, respectively.

Supplementary Figure 2. Page 5

The differentially abundant proteins (DAPs) modulated in *A. fumigatus* upon interaction with *K. pneumoniae* and the up- and downregulated biological enrichment processes according to GSEA analysis. a, Total numbers of proteins present in the proteomes of *A. fumigatus* (reference strain Af293) and *K. pneumoniae* (ATCC 700603) (protein counts retrieved by uniprot.org/proteomes, assessed on 10th April 2020). The number of proteins detected by MS and the correspondent values of mapped proteins in %. **b**, Volcano plot representing the up- and downregulated proteins, using a log2FC 0.5 cut off and FDR<0.05. **c**, GSEA analysis covering the biological process: carboxylic acid metabolic process within the upregulated biological process and the macromolecule biosynthesis within the downregulated process. Up- and downregulated proteins are shown by red and blue colors, respectively.

Supplementary Figure 3. Page 6

Selected attributes related to metabolomic analysis. Pathway enrichment analysis and topology analysis of metabolites differentially regulated under BFI conditions are displayed. **a**, Scatter plot of downregulated metabolites produced by *A. fumigatus* (Afu). **b**, Bar graph depicting a general overview of Afu-downregulated metabolites within each pathway. **c**, Scatter plot of upregulated metabolites produced by Afu. **d***,* Bar graph of Afu-upregulated metabolites. **e***,* Scatter plot of up- and downregulated metabolites produced by *K. pneumoniae* (Kp) under BFI conditions. **f**, Bar graph of up- and downregulated metabolites of *K. pneumoniae* within each pathway. The node size in the scatter plots reflects the importance of the individual metabolites within the respective pathway (impact), and the colors red, orange, yellow, and white represent high, medium, low, and near zero log-10 (p) values, respectively. Annotated metabolites within the log2FC ±1.0 cutoff were subjected to functional categorization in MetaboAnalyst 5.0 across KEGG pathways, followed by a hypergeometric test. Blue and green numbers placed next to the graph bars correspond with individual pathway nodes in the scatter plot for *A. fumigatus* and *K. pneumoniae*, respectively.

Supplementary Figure 4. Page 7

Overlapping of main biological processes modulated in *A. fumigatus* upon interaction with *K. pneumoniae*. **a**, Schematic representation of genes and processes entwined between transcriptomics and proteomics data. **b**, The most representative biological processes among transcriptomic and proteomic clusters.

Supplementary Figure 5. Page 8

Validation of OMICS data. Genes associated with alternative metabolic pathways and stress responses upon BFI were validated in *A. fumigatus* by RT-qPCR. **a**, The transcription levels of genes belonging to oxidative stress response (*gst, sod1* and *cat2)*, sporulation (*blrA*), sulfur metabolism (*metR*), beta-oxidation (*pox1* and *ctf1*), ethanol metabolism (*pdcA and aldA*), and gluconeogenesis (*fbp1* and *pck-acuF*) are displayed in the upper panel. The transcription levels are represented as relative fold change expression values under BFI conditions compared with the single-pathogen biofilm of *A. fumigatus* as a control. Data are normalized by *b-tub* gene. The values shown represent three biological replicates. Statistical significance was determined using unpaired t-test. **b**, The validated genes belonging to some of the main routes activated to rewire *A. fumigatus* metabolism are highlighted in the bottom panel, such as gluconeogenesis, PPP, fatty acid oxidation, and ethanol metabolism. The validated genes are shown in red rectangles. A question mark indicates the putative regulation of PPP by the transcription factor MetR.

Supplementary Table 1. Page 9

Carbon metabolism and acetyl-CoA supply routes modulated in *A. fumigatus* in response to *K. pneumonia* interaction

Supplementary Table 2. Page 11

The 20 major upregulated and downregulated genes during fungal-bacterial interaction in *A. fumigatus*

Supplementary Table 3. Page 12

The 20 major upregulated and downregulated genes during fungal-bacterial interaction in *K. pneumoniae*

Supplementary Table 4. Page 13

The 20 major upregulated and downregulated proteins during fungal-bacterial interaction in *A. fumigatus*

Supplementary Table 5. Page 14

The 20 major upregulated and downregulated proteins during fungal-bacterial interaction in *K. pneumoniae*

Supplementary Table 6. Page 15

The major upregulated and downregulated metabolites in *A. fumigatus* (left side) and *K. pneumoniae* (right side) upon BFI interaction

Supplementary Table 7. Page 16

Integration of transcriptomics and proteomics within the upregulated cluster

Supplementary Table 8. Page 17

Integration of transcriptomics and proteomics within the downregulated cluster

Supplementary Table 9. Page 19

Routes involved in sulfate metabolism modulated in *A. fumigatus* during its interaction with *K. pneumoniae*

Supplementary Table 10. Page 20

Set of oligonucleotides used for qPCR assay

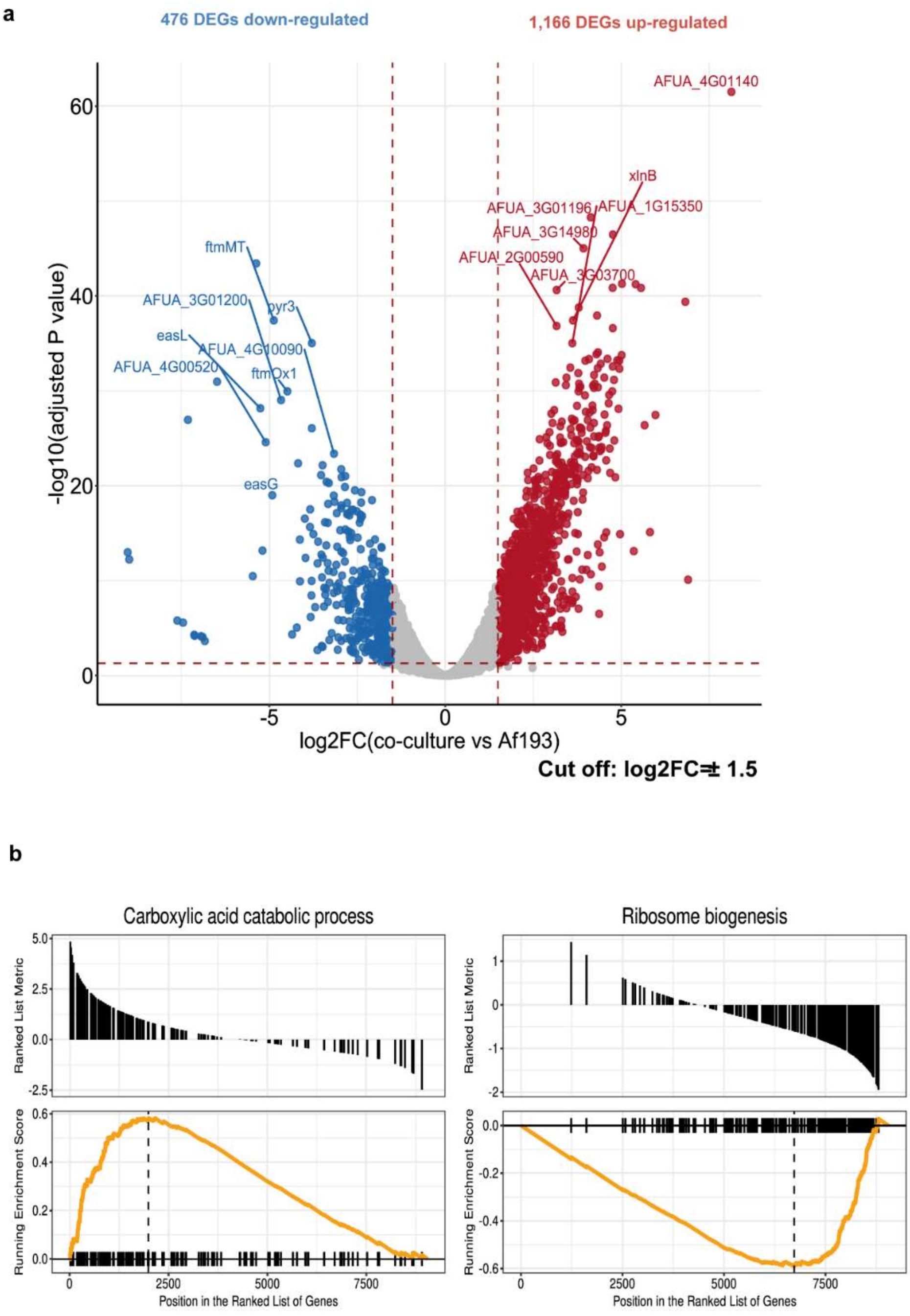

Supplementary Figure 1. Overview of the differentially expressed genes (DEGs) in *A. fumigatus* under bacterial-fungal interaction conditions and the enriched modulated processes according to GSEA enrichment analysis. a, Volcano plot representing the up- and downregulated DEGs is shown, using log2FC±1.5 cut off and p<0.05, upon BFI. **b**, GSEA analysis covering the biological process: carboxylic acid catabolism and ribosome biogenesis determined by transcriptomic analysis. Up- and downregulated genes are shown by red and blue colors, respectively.

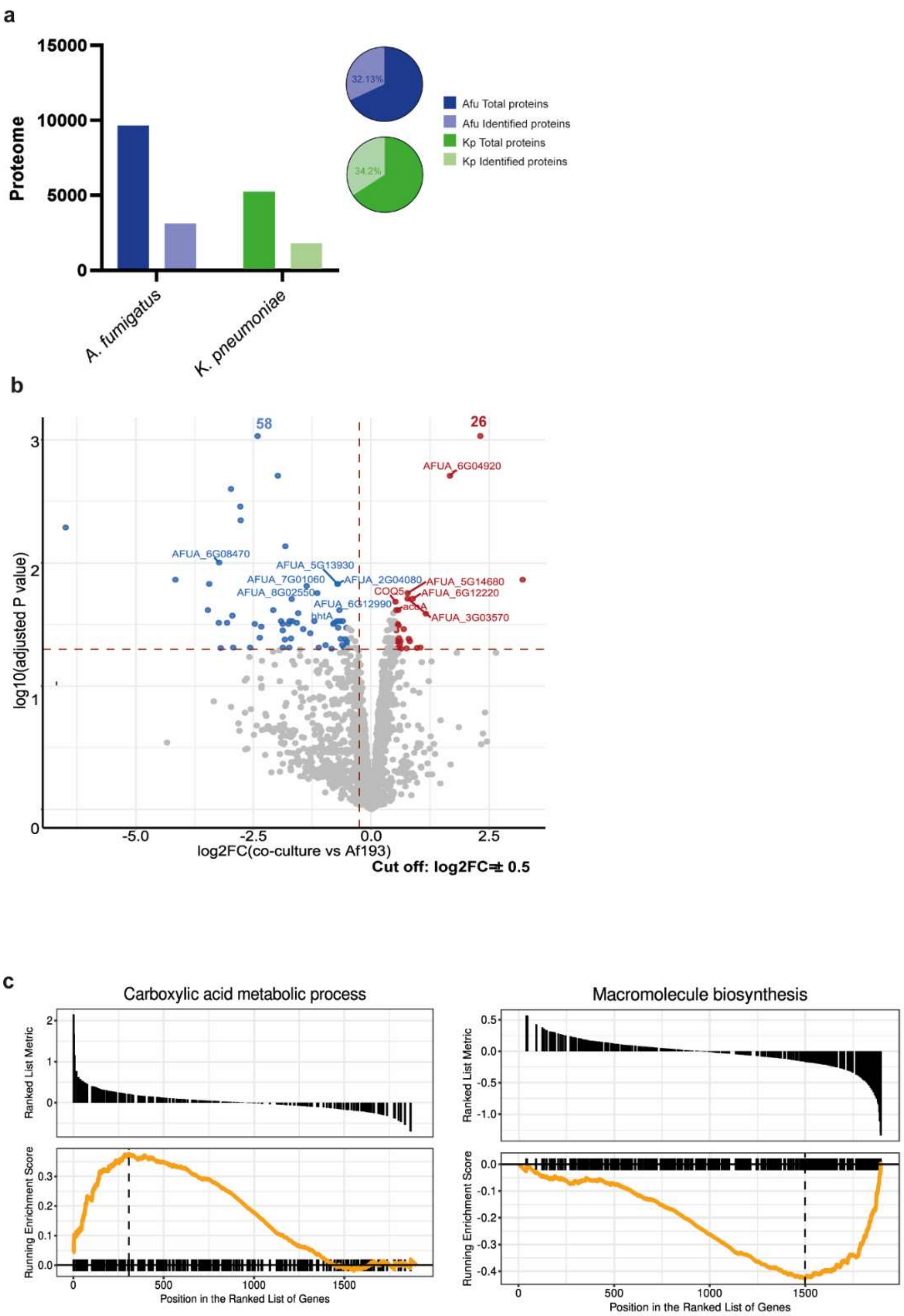

Supplementary Figure 2. The differentially abundant proteins (DAPs) modulated in *A. fumigatus* upon interaction with *K. pneumoniae* and the up- and downregulated biological enrichment processes according to GSEA analysis. a, Total numbers of proteins present in the proteomes of *A. fumigatus* (reference strain Af293) and *K. pneumoniae* (ATCC 700603) (protein counts retrieved by uniprot.org/proteomes, assessed on 10th April 2020). The number of proteins detected by MS and the correspondent values of mapped proteins in %. **b**, Volcano plot representing the up- and downregulated proteins, using a log2FC 0.5 cut off and FDR<0.05. **c**, GSEA analysis covering the biological process: carboxylic acid metabolic process within the upregulated biological process and the macromolecule biosynthesis within the downregulated process. Up- and downregulated proteins are shown by red and blue colors, respectively.

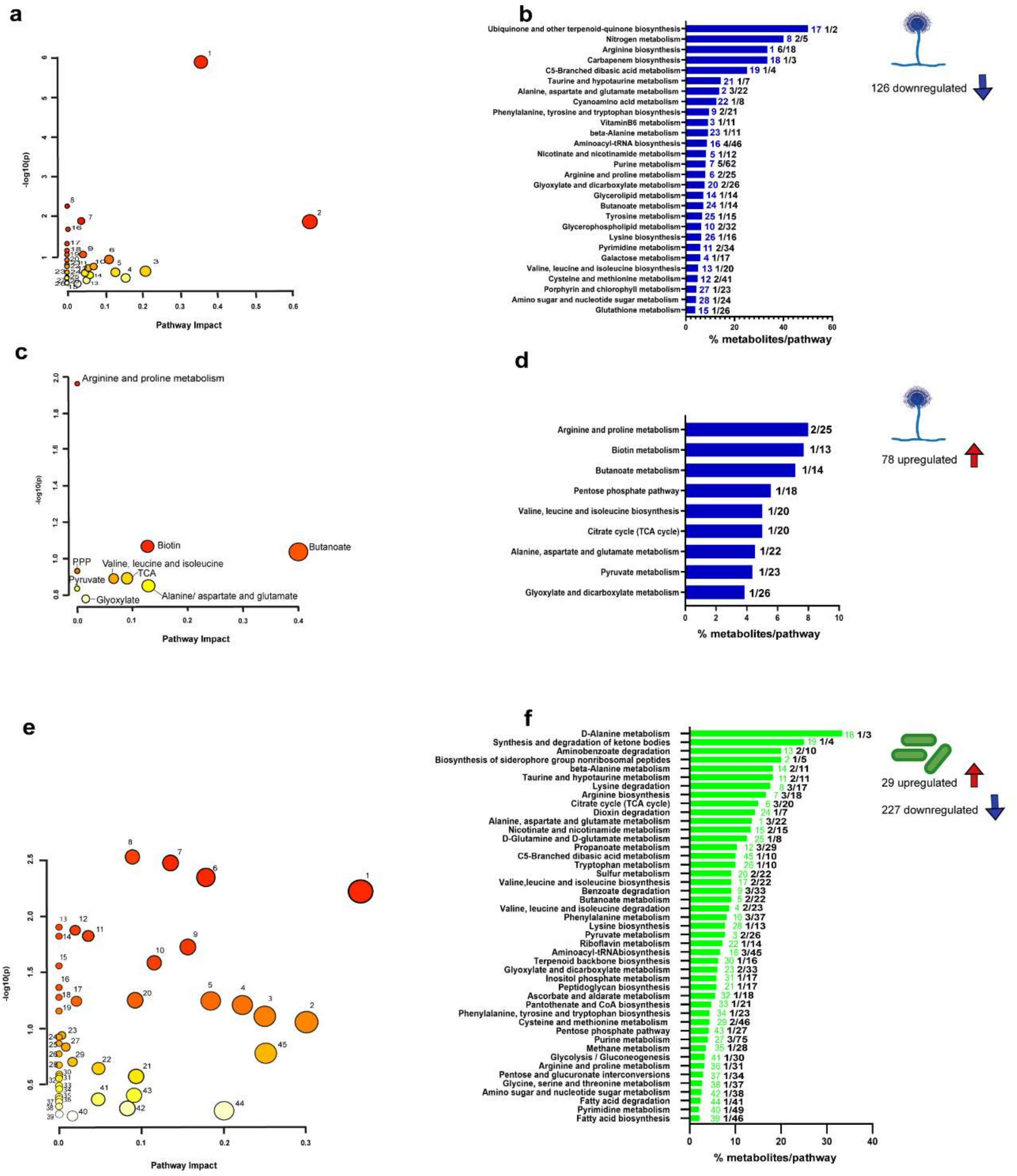

Supplementary Figure 3. Selected attributes related to metabolomic analysis. Pathway enrichment analysis and topology analysis of metabolites differentially regulated under BFI conditions are displayed. **a**, Scatter plot of downregulated metabolites produced by *A. fumigatus* (Afu). **b**, Bar graph depicting a general overview of Afu-downregulated metabolites within each pathway. **c**, Scatter plot of upregulated metabolites produced by Afu. **d**, Bar graph of Afu-upregulated metabolites. **e**, Scatter plot of up- and downregulated metabolites produced by *K. pneumoniae* (Kp) under BFI conditions. **f**, Bar graph of up- and downregulated metabolites of *K. pneumoniae* within each pathway. The node size in the scatter plots reflects the importance of the individual metabolites within the respective pathway (impact), and the colors red, orange, yellow, and white represent high, medium, low, and near zero log-10 (p) values, respectively. Annotated metabolites within the log2FC ±1.0 cutoff were subjected to functional categorization in MetaboAnalyst 5.0 across KEGG pathways, followed by a hypergeometric test. Blue and green numbers placed next to the graph bars correspond with individual pathway nodes in the scatter plot for *A. fumigatus* and *K. pneumoniae*, respectively.

a

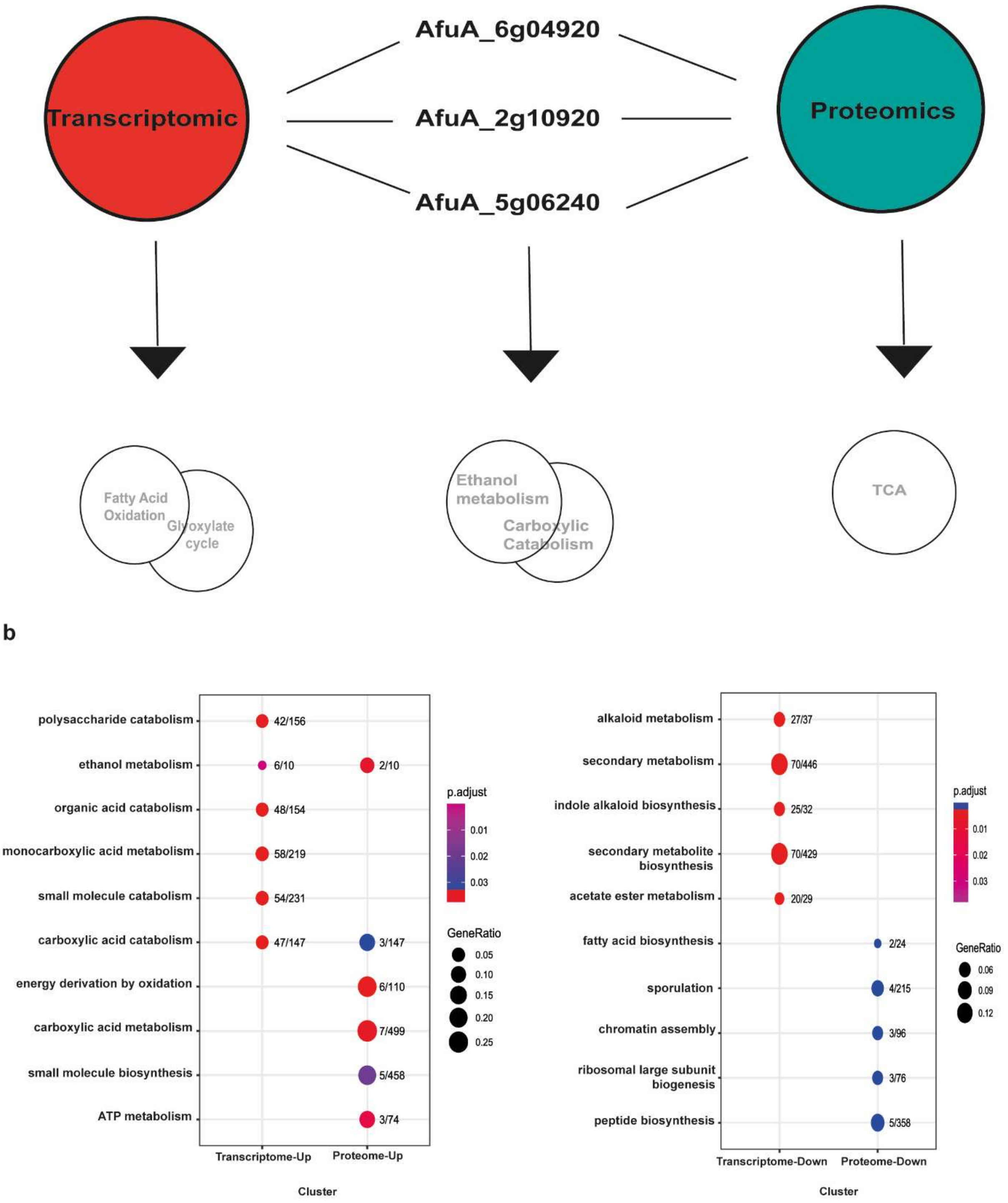

Supplementary Figure 4. Overlapping of main biological processes modulated in *A. fumigatus* upon interaction with *K. pneumoniae*. a, Schematic representation of genes and processes entwined between transcriptomics and proteomics data. **b,** The most representative biological processes among transcriptomic and proteomic clusters.

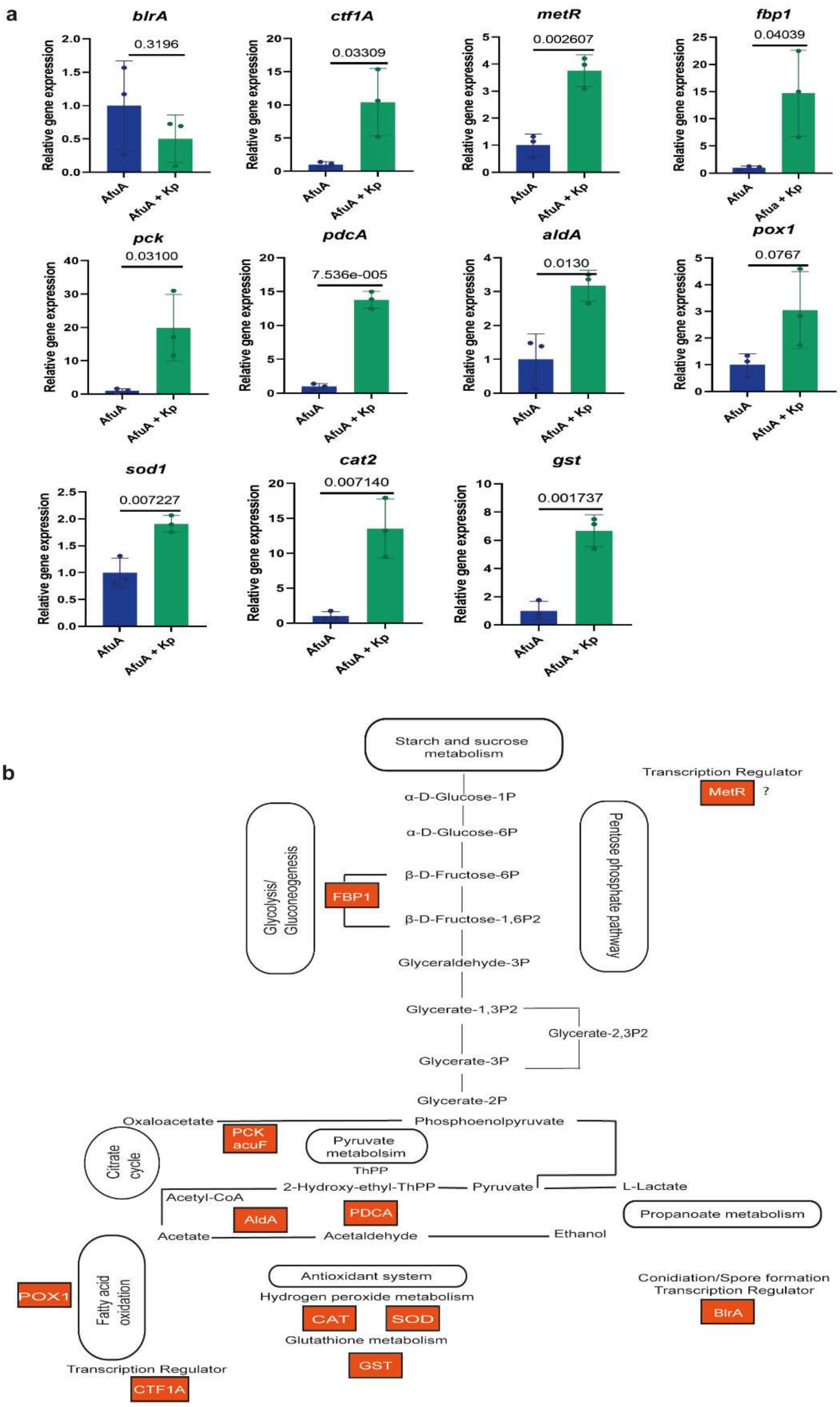

Supplementary Figure 5. Validation of OMICS data. Genes associated with alternative metabolic pathways and stress responses upon BFI were validated in *A. fumigatus* by RT-qPCR. **a**, The transcription levels of genes belonging to oxidative stress response (*gst, sod1* and *cat2)*, sporulation (*blrA*), sulfur metabolism (*metR*), beta-oxidation (*pox1* and *ctf1*), ethanol metabolism (*pdcA and aldA*), and gluconeogenesis (*fbp1* and *pck-acuF*) are displayed in the upper panel. The transcription levels are represented as relative fold change expression values under BFI conditions compared with the single-pathogen biofilm of *A. fumigatus* as a control. Data are normalized by *b-tub* gene. The values shown represent three biological replicates. Statistical significance was determined using unpaired t-test. **b**, The validated genes belonging to some of the main routes activated to rewire *A. fumigatus* metabolism are highlighted in the bottom panel, such as gluconeogenesis, PPP, fatty acid oxidation, and ethanol metabolism. The validated genes are shown in red rectangles. A question mark indicates the putative regulation of PPP by the transcription factor MetR.

Supplementary Table 1. Carbon metabolism and acetyl-CoA supply routes modulated in *A. fumigatus* in response to *K. pneumonia* interaction

Gene/Protein	Identifier	Cluster Data	LOG2FC
Lipid Metabolism			
3-oxoacyl-acyl carrier protein reductase	AfuA_6G10520	Proteomics/Transcript	+1.029/-0.68
Phospholipase PldA	AfuA_7G05580	Proteomics/Transcript	+0.480/+2.07
Phospholipase A2-like	AfuA_2g11970	Transcript	+1.65
Putative extracellular lipase	AfuA_8g02530	Transcript	+3.81
Triglyceride lipase activity	AfuA_7g04950	Transcript	+2.64
Putative phospholipase D	AfuA_3g05630	Transcript	+1.96
Ethanol Metabolism			
Pyruvate decarboxylase (PdcA)	AfuA_3g11070	Proteomics/Transcript	+0.58/+4.65
Pyruvate decarboxylase	AfuA_2g01040	Transcript	+1.77
Aldehyde dehydrogenase AldA	AfuA_6g11430	Transcript	+1.93
Putative alcohol dehydrogenase	AfuA_7g01000	Proteomics/Transcript	+0.852/+3.14
Alcohol dehydrogenase<br>Alcohol dehydrogenase	AfuA_7g01010<br>AfuA_5g06240	Transcript<br>Proteomics/Transcript	+3.29<br>+1.158/+4.20
Ortholog(s) have aldehyde dehydrogenase	AfuA_6g10120	Transcript	+2.42
Putative aldehyde dehydrogenase	AfuA_6g11430	Transcript	+1.93
Pentose Phosphate Pathway			
Putative 6-phosphogluconolactonase	AfuA_1g02980	Transcript	+2.43
Phosphogluconate dehydrogenase (NAD/NADP)	AfuA_6g08730	Transcript	+3.16
Phosphogluconate dehydrogenase (NAD/NADP)	AfuA_5g10280	Transcript	+2.17
Phosphogluconate dehydrogenase (NAD/NADP)	AfuA_5g01250	Transcript	+1.80
TCA cycle			
Succinate dehydrogenase	AfuA_1g15590	Transcript	+0.70
Succinate dehydrogenase	AfuA_5g09680	Transcript	+1.00
Succinate dehydrogenase	AfuA_3G07810	Proteomics/Transcript	+0.58/+0.72
Citrate synthase	AfuA_5g04230	Transcript	+0.52
Aconitate hydratase	AfuA_6g12930	Proteomics/Transcript	+0.53/+0.79
Putative isocitrate dehydrogenase	AfuA_3g08660	Transcript	+0.85
NAD(+)-isocitrate dehydrogenase subunit I	AfuA_6g06370	Transcript	+0.55
Succinyl-CoA synthetase	AfuA_4g00290	Transcript	+2.00
Fumarate reductase	AfuA_8g05530	Transcript	+2.61
Malate dehydrogenase_SrbA	AfuA_7g02420	Transcript	+1.52
Malate dehydrogenase	AfuA_7G05740	Proteomics	+0.62
Malate dehydrogenase	AfuA_6g05210	Transcript	+0.72
Alpha-ketoglutarate	AfuA_3g07960	Transcript	+1.54
Putative mitochondrial carnitine	AfuA_6g14100	Transcript	+1.58
Putative carnitine acetyl transferase	AfuA_2g12530	Transcript	+1.83
Putative carnitine acetyl transferase	AfuA_1g12340	Transcript	+2.00
Putative alpha-ketoglutarate dehydrogenase	AfuA_4g11650	Proteomics/Transcript	+0.186/+1.15
Glyoxylate Metabolism			
Malate synthase	AfuA_6g03540	Proteomics/Transcript	-0.70/+3.22
Putative isocitrate lyase	AfuA_4g13510	Transcript	+4.83
GABA shunt			
Putative glutamate dehydrogenase	AfuA_4g06620	Transcript	-0.71
NAD+ dependent glutamate dehydrogenase	AfuA_2g06000	Transcript	+2.49
Putative glutamate decarboxylase	AfuA_6g13490	Transcript	+2.90
Putative succinate-semialdehyde dehydrogenase	AfuA_3g07150	Transcript	+0.62
4-aminobutyrate aminotransferase	AfuA_5g06680	Transcript	-0.76
Gluconeogenesis Metabolism			
Putative phosphoenolpyruvate carboxy kinase (Pck1)	AfuA_6g07720	Transcript	+3.18
Fructose-1,6-bisphosphatase (Fbp)	AfuA_4g11310	Transcript	+3.02
β-oxidation of Fatty Acids			
Enoyl-CoA hydratase	AfuA_2g10920	Proteomics/Transcript	+0.53/+1.39
Fatty-acyl CoA oxidase (Pox1)	AfuA_7g06090)	Proteomics/Transcript	-1.91/2.48
Acyl-CoA dehydrogenase	AfuA_1g14850	Transcript	+1.71
Acyl-CoA dehydrogenase	AfuA_7g06100	Transcript	+2.18
Ketoacyl-CoA ketothiolase (Kat1)	AfuA_1g12650	Transcript	+1.92
Ketoacyl-CoA ketothiolase	AfuA_4g10950	Transcript	+1.45
C6 transcription factor (Cat8)	AfuA_1g13510	Transcript	+1.71
Acetyl COA and others			

Putative carnitine acetyl transferase	AfuA_1g12340	Transcript	+2.0
Putative carnitine acetyl transferase	AfuA_2g12530	Transcript	+1.85
Acetyl-CoA acetyltransferase	AfuA_6G14200	Transcript	+3.03
Acetyl-CoA hydrolase Ach1	AfuA_8G05580	Proteomics/Transcript	+2.647/3.79
Putative acetoacetyl-CoA synthase	AfuA_8g04770	Transcript	+1.69
Glucose repressible protein Grg1	AfuA_5G14210	Proteomics/Transcript	+3.215/+4.33
Carbon and Cell Wall biosynthesis			
Putative alpha(1-3) glucan synthase	AfuA_1g15440	Transcript	+1.69
Cell wall glucanase; allergen Asp f 9	AfuA_1g16190	Transcript	+2.12
Essential 1,3-beta-glucanosyltransferase	AfuA_2g05340	Transcript	+3.01
Ortholog(s) have glucan endo-1,6-beta-glucosidase activity, catabolic processes	AfuA_2g09350	Transcript	+3.75
GPI-anchored endo-beta-1,3-glucanase	AfuA_2g14360	Transcript	+2.42
Ortholog(s) have cellulase activity	AfuA_2g14540	Transcript	+1.81
Cell wall glucanase	AfuA_3g00270	Transcript	+2.04
Novel beta-1,3-glucan modifying enzyme	AfuA_7g05450	Transcript	+2.03
Putative secreted 1,4-beta-D-glucan glucanhydrolase	AfuA_7g06140	Transcript	-2.93
Putative alpha-1,3-glucanase	AfuA_8g06360	Transcript	-2.90
Putative endo-1,4 beta-glucanase	AfuA_8g06830	Transcript	+2.57
Class III chitinase ChiA2	AfuA_8g00700	Transcript	+1.92
Class III chitinase ChiA1	AfuA_5g03760	Transcript	+2.05
Class V chitinase, putative	AfuA_7g08490	Transcript	+1.89

+/-numbers referred to up- and down-regulated genes and/or proteins

Supplementary Table 2. The 20 major upregulated and downregulated genes during fungal-bacterial interaction in *A. fumigatus*

Gene_ID	Gene Description	LOG2FC	Biological Process
AfuA_4g01140	MFS multidrug transporter, putative	+8.13	Transmembrane transport
AfuA_5g00310	flavin-containing monooxygenase, putative	+6.90	Oxidation-reduction process
AfuA_8g01630	pyridine nucleotide-disulphide oxidoreductase protein	+6.82	Oxidation-reduction process
AfuA_4g14712	C6 transcription factor, putative	+5.97	Transcription
AfuA_5g01470	peroxisomal copper amine oxidase	+5.82	Metabolic processes
AfuA_3g03180	NAD binding Rossmann fold oxidoreductase, putative	+5.66	Metabolic processes
AfuA_6g09740	thioredoxin reductase GliT	+5.56	Secondary metabolism
AfuA_6g09730	cytochrome P450 oxidoreductase GliF	+5.41	Secondary metabolism
AfuA_5g00300	zinc-binding oxidoreductase	+5.36	Oxidation-reduction process
AfuA_8g06554	carbonate dehydratase, putative	+5.02	Oxidative stress response
AfuA_2g09330	C6 finger domain protein, putative	+5.01	Transcription
AfuA_2g00630	GDSL lipase/acylhydrolase family protein	+4.97	Metabolic processes
AfuA_3g12190	RING finger domain protein, putative	+4.95	Transcription
AfuA_3g12740	copper resistance-associated P-type ATPase, putative	+4.95	Metabolic processes
AfuA_8g05600	conserved hypothetical protein	+4.92	NA
AfuA_3g00670	DUF1275 domain protein	+4.89	NA
AfuA_4g13510	isocitrate lyase acuD	+4.83	Carbon utilization
AfuA_8g01690	conserved hypothetical protein	+4.80	NA
AfuA_5g09970	myosin-cross-reactive antigen family protein	+4.79	Fatty acid metabolic process
AfuA_3g13620	cupin domain protein	+4.76	Secondary metabolism
AfuA_8g06090	amino acid permease, putative	-9.03	Metabolic processes
AfuA_2g18020	O-acetyltransferase, putative	-8.98	Secondary metabolism biosynthesis
AfuA_2g17980	cytochrome P450 monooxygenase,	-7.62	Secondary metabolism biosynthesis
AfuA_6g09530	hypothetical protein	-7.45	NA
AfuA_8g00220	cytochrome P450, putative	-7.31	Secondary metabolism biosynthesis
AfuA_5g06180	predicted DDE1 transposon-related ORF	-7.13	NA
AfuA_6g12140	hypothetical protein	-7.12	NA
AfuA_7g06840	class III aminotransferase, putative	-6.94	Metabolic processes
AfuA_4g13830	MFS multidrug transporter, putative	-6.89	Transport
AfuA_1g17210	conserved hypothetical protein	-6.83	NA
AfuA_8g00210	dimethylallyl tryptophan synthase FtmPT1	-6.49	Secondary metabolism biosynthesis
AfuA_2g09400	cyclohexanone monooxygenase	-5.47	Oxidation-reduction process
AfuA_6g11850	hypothetical protein	-5.37	NA
AfuA_2g17990	dimethylallyl tryptophan synthase FgaPT1	-5.26	Alkaloid metabolism
AfuA_2g17160	hypothetical protein	-5.19	NA
AfuA_4g00520	hypothetical protein	-5.10	NA
AfuA_2g17970	ergot alkaloid biosynthetic protein A	-4.92	Alkaloid biosynthetic process
AfuA_8g00200	O-methyltransferase, putative	-4.87	Fumitremorgin B biosynthetic process
AfuA_3g01200	integral membrane protein Pth11-like, putative	-4.67	NA
AfuA_8g00230	phytanoyl-CoA dioxygenase family protein	-4.49	Verruculogen biosynthesis

+/-numbers referred to up- and down-regulated genes

Supplementary Table 3. The 20 major upregulated and downregulated genes during fungal-bacterial interaction in *K. pneumoniae*

GeneID	Gene Description	LOG2FC	Biological Process
gene3907	D-ribose pyranase	+7.28	Carbohydrate metabolism
gene3849	6-phospho-alpha-glucosidase	+6.59	Carbohydrate metabolism
gene2129	ribitol 2-dehydrogenase	+5.69	Oxireductase process
gene4243	AraC family transcriptional regulator	+5.66	Transcription
gene4168	malate synthase A	+5.33	Carbon metabolism
gene3908	ribose ABC transporter ATP-binding protein RbsA	+5.06	Sugar transport
gene4396	hypothetical protein/DUF1107 domain-containing protein	+4.78	Transcription
gene237	DNA starvation/stationary phase protection protein	+4.78	DNA protection during starvation
gene256	glycyl-radical enzyme activating protein family	+4.73	Oxireductase process
gene5653	hypothetical protein	+4.71	NA
gene3850	PTS alpha-glucoside transporter subunit IICB	+4.69	Carbohydrate metabolism
gene1536	hypothetical protein	+4.69	NA
gene5059	PTS mannose transporter subunit IIA	+4.55	Carbohydrate transport
gene1120	heat-shock protein 20	+4.50	Stress response
gene3763	aquaporin	+4.46	Transport
gene4731	hypothetical protein/Glucose uptake inhibitor SgrT	+4.39	Sugar transport
gene5147	50S ribosomal protein L31	+4.38	Translation
gene2174	methyl-galactoside ABC transporter substrate-binding protein	+4.36	Carbohydrate transport
gene5148	50S ribosomal protein L36	+4.27	Translation
gene5586	hypothetical protein/CopG family helix-turn-helix protein	+4.24	Transcription
gene2949	hypothetical protein	-8.53	NA
gene2464	phosphate acetyltransferase	-5.10	NA
gene4282	phosphonate C-P lyase system protein PhnG	-4.93	Phosphate transport
gene1611	cytochrome c-type biogenesis protein CcmC heme lyase for CcmE	-4.91	ABC transport
gene4497	hypothetical protein/DUF2878	-4.83	NA
gene5018	taurine ABC transporter ATP-binding protein	-4.78	ATP-binding
gene2778	hypothetical protein	-4.67	NA
gene2260	2-succinylbenzoate-CoA ligase	-4.66	Menaquinone biosynthesis
gene2187	1-phosphofructokinase	-4.65	Sugar metabolism
gene1591	acetyl-CoA acetyltransferase	-4.59	Lipid homeostasis
gene2895	NA	-4.58	
gene192	8-amino-7-oxononanoate synthase	-4.58	Biotin biosynthesis
gene3503	hypothetical protein	-4.52	NA
gene2896	NA	-4.35	NA
gene2186	PTS fructose transporter subunit EIIBC	-4.32	Sugar transport
gene4009	hypothetical protein	-4.25	NA
gene1612	heme exporter protein CcmB	-4.23	Cytochrome assembly
gene5002	ABC transporter	-4.20	ATP-binding
gene1146	branched-chain amino acid ABC transporter permease	-4.16	Transport
gene5355	peptide ABC transporter permease	-4.12	Transport

+/-numbers referred to up- and down-regulated genes

Supplementary Table 4. The 20 major upregulated and downregulated proteins during fungal-bacterial interaction in *A. fumigatus*

Protein ID	Gene Description	LOG2FC	Biological Process
A4D9A5	uncharacterized protein	+2.316	Mitochondrial function
Q4WW28	glucose repressible protein Grg1	+3.215	Carbohydrate regulator
Q4WDJ0	formate dehydrogenase	+1.670	Formate catabolic process
Q4WTV5;Q4WAE4	alcohol dehydrogenase	+1.158	Ethanol metabolism
Q4WCH3	mRNA cleavage and polyadenylation complex subunit (Pta1)	+1.044	mRNA polyadenylation
Q4WLJ3	RNA binding protein	+0.972	NA
Q4WLV1	isochorismatase family hydrolase	+0.880	Hydrolase activity
Q4WXU7	probable acetate kinase	+0.818	Acetyl-CoA biosynthesis
Q4WQ64	phosphatidyl synthase	+0.796	Glycerophospholipid biosynthesis
Q4WW75	uncharacterized protein	+0.773	NA
Q4WF38	67 kDa myosin-cross-reactive antigen family protein	+0.772	Fatty acid metabolism
Q4WMF5	uncharacterized protein	+0.750	Transferase activity
Q4WDH8	lysine decarboxylase-like protein	+0.690	Response to stimuli
O60024	allergen Asp f 4	+0.627	NA
Q4WGP3	malate dehydrogenase	+0.620	TCA cycle
Q4WP70	zinc-containing alcohol dehydrogenase	+0.603	Alcohol metabolism
Q4X1L6	DUF833 domain protein	+0.588	Golgi organization
Q4WX09	succinate dehydrogenase	+0.579	TCA cycle
Q4WXX9	pyruvate decarboxylase	+0.577	Ethanol metabolism
Q4WU09	carbamoyl-phosphate synthase arginine-specific	+0.576	Amino acid metabolism
Q4W9N5	AP-2 adaptor complex subunit alpha	-2.334	Endocytosis
Q4WQ48	vacuolar protein sorting-associated protein (VPS13)	-6.488	Ascospore formation
Q4WJD0	nuclear localization protein NPL6	-4.158	Chromatin remodeling
Q4X070	2-dehydropantoate 2-reductase	-3.468	Pantothenate biosynthesis
Q4WJA8	golgi phosphoprotein 3 (GPP34)	-3.440	Vesicle-mediated transport
Q4WGT3	probable beta-glucosidase	-3.238	Glucan catabolism
Q4WMW6	glycerol kinase	-3.229	Glycerol metabolism
Q4WXS1	DNA damage response protein	-3.195	Ergosterol biosynthesis
Q4WS51	uncharacterized protein	-3.060	NA
Q4WVI3	phosducin	-2.977	Signaling pathway
Q4X1L4	biotin apo-protein ligase	-2.949	Protein modification
Q4WU45	vacuolar protein sorting-associated protein 17	-2.930	Vesicle-mediated transport
Q4WCH1	COP9 signalosome subunit 8 (CsnH)	-2.779	Protein deneddylation
Q4WYE0	DUF250 domain membrane protein	-2.772	Antiporter
Q4WZI1	sister chromatid cohesion and DNA repair protein (BimD)	-2.571	Cell division
Q4WJ49	DSBA-like thioredoxin domain protein	-2.473	Oxidation reduction
Q4WJP7	PHD finger and BAH domain protein	-2.413	Transcription regulation
Q4WCH5	nucleolar protein 12	-2.367	rRNA processing
Q4WA11	got1 family protein	-2.081	Vesicle-mediated transport
Q4WZV4	DUF92 domain protein	-1.983	NA

+/-numbers referred to up- and down-regulated proteins

Supplementary Table 5. The 20 major upregulated and downregulated proteins during fungal-bacterial interaction in *K. pneumoniae*

Uniprot Ac. Number	Gene Description	LOG2FC	Biological Process
M7PCH5	high-affinity branched-chain amino acid transporter periplasmic binding component	+5.179	Amino acid transport
M7Q816	ABC transport system periplasmic binding component	+5.096	Transport
M7PFS7	50S ribosomal protein L33	+3.120	Translation
M7Q2A7	ATP-dependent RNA helicase DeaD	+3.006	Response to stress
M7QBQ4	adenosylmethionine--8-amino-7-oxononanoate transaminase	+2.897	Biotin /sulfur metabolism
M7QL09	glutamate and aspartate transporter subunit	+2.728	Transport
M7P9K0	RND family efflux transporter MFP subunit	+2.724	Transport
M7P9X0	hypothetical protein	+2.600	NA
M7P7T1	50S ribosomal protein L17	+2.448	Translation
M7PYG6	30S ribosomal protein S19	+2.359	Translation
M7PWY7	cold shock protein/RNA chaperone	+2.152	Response to stress
M7QL87	kinase inhibitor protein	+2.089	Kinase/Sensing
M7PUJ9	putrescine transporter subunit: periplasmic-binding component of ABC superfamily	+2.058	Amino transport
M7PYK9	ABC transporter lysine/arginine/ornithine binding periplasmic protein	+2.056	Nitrogen transport
M7PCB5	nitrogen regulatory protein P-II 2	+2.045	Nitrogen metabolism
M7QQC5	ABC transporter arginine-binding protein	+1.993	Amino acid transporter
M7PZC3	lipoprotein	+1.983	NA
M7PKI4	50S ribosomal protein L32	+1.879	Translation
M7PSN8	siderophore-interacting protein	+1.879	Oxidoreductase
M7PZH9	5-methyltetrahydropteroyltriglutamate--homocysteine S-methyltransferase	+1,878	Methionine metabolism
M7PRW4	acetolactate synthase	-8.133	Carboxilic acid metabolism
M7Q8M4	alpha-acetolactate decarboxylase	-7.045	Carbon metabolism
M7P5U7	excinuclease ABC subunit A	-6.616	Response to stress
M7PLX4	acetolactate synthase 3 catalytic subunit	-6.603	Metabolism
M7PYC4	malate:quinone oxidoreductase	-6.588	TCA metabolism
M7PH71	preprotein translocase subunit SecD	-6.583	Transport
M7PNH6	translation elongation factor G	-6.517	Translation
M7QL44	2-oxoglutarate dehydrogenase E1 component	-6.440	Carbon metabolism/TCA
M7PZG6	cell division protein MukB	-6.368	Cell division
M7PB41	valyl-tRNA ligase	-6.190	Protein biosythesis
M7P7W1	Small heat shock protein	-6.122	Response to stress
M7QKG2	iron ABC transporter substrate-binding protein	-6.056	Iron transport
M7QCY9	transcriptional accessory protein	-5.980	transcription
M7QQ92	D-alanyl-D-alanine carboxypeptidase fraction C	-5.944	Cell wall organization
M7PRV9	siroheme synthase	-5.931	Cobalamin biosynthesis
M7P8V7	acetolactate synthase 2 catalytic subunit	-5.912	Amino acid metabolism
M7QML4	ABC transporter ATP-binding protein	-5.833	transport
M7QCC1	membrane protein insertase	-5.735	Membrane component
M7Q4W2	carbon storage regulator CsrA	-5.703	Carbohydrate metabolism
M7Q5D5	B12-dependent methionine synthase	-5.684	Carbon Metabolism

+/-numbers referred to up- and down-regulated proteins

Supplementary Table 6. The major upregulated and downregulated metabolites in *A. fumigatus* (left side) and *K. pneumoniae* (right side) upon BFI interaction

**Metabolites (BFI x AfuA)**	**LOGFC (BFI x AfuA)**	**Metabolites (BFI x Kp)**	**LOGFC (BFI x Kp)**
Ascorbic acid	+6.09	6-Methylnicotinamide	+3.68
Norfenefrine-like	+6.07	α-D-Mannose 1-phosphate	+2.87
2-Hydroxycaproic acid	+5.86	L-Aspartic acid	+1.45
2-Deoxyribose 5-phosphate	+5.79	Methamphetamine	+1.30
2-Isopropylmalic acid	+5.71	Amphetamine	+1.15
Nipecotic acid	+2.25	Gluconic acid	+1.04
Desthiobiotin	+1.64	Pipecolic acid	0.66
Citric acid	+1.36	Mevalonic acid	0.58
4-Acetamidobutanoic acid	+1.25	Fumaric acid	0.55
Amphetamine	+1.15	Acadesine	0.28
Y-Aminobutyric acid (GABA)	+1.13	DL-Dihydroorotic acid	0.26
Glycerophospho-N-palmitoyl ethanolamine	+1.13	2-Hydroxyvaleric acid	0.25
Uracil	-6.56	L-Phenylalanine	-5.21
Hippuric acid	-5.6	Imidazoleacetic acid	-4,93
Xanthine	-5.12	Cytosine	-4.80
4-Hydroxyphenylpyruvic acid	-4.06	7-Methylguanine	-4.68
Mevalonolactone	-2.80	N-Acetylornithine	-4.60
Levulinic acid	-2.04	Adenosine 3'5'-cyclic monophosphate	-4.29
N-Acetylvaline	-1.86	N-Acetyl-L-tyrosine	-4.18
Hypoxanthine	-1.81	2,3-Dihydroxybenzoic acid	-3,83
Indole-3-acrylic acid	-1.77	Neopterin	-3.75
Propionylcarnitine	-1.73	Acetyl-CoA	-3.44
2'-O-Methylguanosine	-1.72	Quinic acid	-3.25
Pyridoxal	-1.54	4-Guanidinobutyric acid	-3.21
N-Acetylornithine	-1.52	N-Acetyl-L-glutamine	-3.19
Picolinic acid	-1.47	L-Threonic acid	-3.17
Nicotinic acid	-1.41	2-Furoic acid	-3.07
N-Acetylornithine	-1.40	Xanthosine	-3.07
1,5-Anhydro-D-glucitol	-1,37	3-Hydroxy-5-(hydroxymethyl)-2-methylisonicotinaldehyde oxime	-3.07
Nicotine	-1.36	Caffeic acid	-3.06
Guanine	-1.32	3-Hydroxybenzoic acid	-2.92
D-(+)-Galactose	-1.31	Uridine 5'-diphosphoglucuronic acid	-2.87

+/-numbers referred to up- and down-regulated metabolites

Supplementary Table 7. Integration of transcriptomics and proteomics within the upregulated cluster

Gene Id	Description	Biological Process
AfuA_3g03570	myosin-cross-reactive antigen family protein	Carboxylic acid metabolism
AfuA_5g06780	carbamoyl-phosphate synthase	Carboxylic acid metabolism
AfuA_6g12930	aconitate hydratase	Carboxylic acid metabolism/energy derivation
AfuA_7g05740	malate dehydrogenase	Carboxylic acid metabolism/energy derivation
AfuA_3g07810	succinate dehydrogenase subunit Sdh1	Energy derivation by oxidation
AfuA_6g08850	ubiquinone biosynthesis methlytransferase Coq5	Energy derivation by oxidation
AfuA_3g11070	pyruvate decarboxylase PdcA	Energy derivation by oxidation
AfuA_2g10920	enoyl-CoA hydratase	Carboxylic acid metabolism/carboxylic acid catabolism
AfuA_5g06240	alcohol dehydrogenase	Carboxylic acid metabolism/energy derivation/ carboxylic acid catabolism
AfuA_6g04920	formate dehydrogenase AciA/Fdh	Carboxylic acid metabolism/carboxylic acid catabolism
AfuA_7g06090	fatty-acyl coenzyme A oxidase (Pox1)	carboxylic acid catabolism
AfuA_5g07510	C6 transcription factor AlcR	Carboxylic acid metabolism/carboxylic acid catabolism
AfuA_1g12340	carnitine acetyl transferase	Carboxylic acid metabolism/carboxylic acid catabolism
AfuA_8g06020	glutamate decarboxylase	Carboxylic acid metabolism/energy derivation/ carboxylic acid catabolism
AfuA_2g04240	maleylacetoacetate isomerase MaiA	Carboxylic acid catabolism
AfuA_6g14200	acetyl-CoA-acetyltransferase	Carboxylic acid catabolism
AfuA_6g11430	aldehyde dehydrogenase AldA	Carboxylic acid catabolism
Afu6g08750	delta-1-pyrroline-5-carboxylate dehydrogenase PrnC	Carboxylic acid catabolism
AfuA_2g06000	NAD+ dependent glutamate dehydrogenase	Carboxylic acid catabolism
AfuA_7g01010	alcohol dehydrogenase	Carboxylic acid catabolism
AfuA_2g11350	peroxisomal 3-ketoacyl-coA thiolase (Kat1)	Carboxylic acid catabolism
AfuA_3g14250	Indoleamine 2,3-dioxygenase	Carboxylic acid catabolism
AfuA_8g01210	enoyl-CoA hydratase	Carboxylic acid catabolism
AfuA_4g03900	peroxisomal multifunctional beta-oxidation protein (MFP)	Carboxylic acid catabolism
AfuA_2g12530	carnitine acetyl transferase	Carboxylic acid catabolism
AfuA_5g01710	cytochrome P450 phenylacetate 2-hydroxylase	Carboxylic acid catabolism
AfuA_8g04130	C6 transcription factor (Ctf1B)	Carboxylic acid catabolism
AfuA_2g13630	aminotransferase Aro8	Carboxylic acid catabolism
AfuA_2g04200	4-hydroxyphenylpyruvate dioxygenase	Carboxylic acid catabolism
AfuA_6g08760	proline oxidase PrnD	Carboxylic acid catabolism
AfuA_2g16930	succinate:fumarate antiporter (Acr1)	Carboxylic acid catabolism
AfuA_6g07770	alanine aminotransferase	Carboxylic acid catabolism
AfuA_1g12650	3-ketoacyl-CoA ketothiolase (Kat1)	Carboxylic acid catabolism
AfuA_2g01040	formaldehyde dehydrogenase	Carboxylic acid catabolism
AfuA_1g14610	lipin Smp2	Carboxylic acid catabolism
AfuA_1g14850	acyl-CoA dehydrogenase	Carboxylic acid catabolism
AfuA_4g13510	isocitrate lyase AcuD	Carboxylic acid catabolism
AfuA_1g13510	C6 transcription factor FacB/Cat8	Carboxylic acid catabolism
AfuA_8g01750	acyl-CoA oxidase	Carboxylic acid catabolism
AfuA_6g02860	isocitrate lyase	Carboxylic acid catabolism
AfuA_6G13490	glutamate decarboxylase	Carboxylic acid catabolism

Supplementary Table 8. Integration of transcriptomics and proteomics within the downregulated cluster

Gene Id	Description	Biological Process
AfuA_3g06070	histone H1	Chromatin modification/assembly
AfuA_2g13860	histone H4.2	Chromatin modification/assembly and sporulation
AfuA_1g13790	histone H3	Chromatin modification/assembly and sporulation
AfuA_6g12990	large ribosomal subunit protein L7A	Ribosomal biogenesis
AfuA_2g13440	chitin synthase ChsE	sporulation
AfuA_1g12530	oleate delta-12 desaturase	Sporulation/fatty acid biosynthesis
AfuA_6g02440	60S ribosomal protein L24a	Ribosomal biogenesis/ peptide biosynthesis
AfuA_1g10510	60S ribosomal protein L35	Ribosomal biogenesis /Peptide biosynthesis
AfuA_5g13930	CCCH finger DNA binding protein	Peptide biosynthesis
AfuA_1g06340	60S ribosomal protein L27e	Peptide biosynthesis
AfuA_4g07435	60S ribosomal protein L36	Peptide biosynthesis
AfuA_8g00190	cytochrome P450	Fumitermorgin B biosynthesis/ 2ndario metabolism/ alkaloid metabolism
AfuA_8g00170	nonribosomal brevianamide peptide synthase FtmA	Fumitermorgin B biosynthesis/ 2ndario metabolism/alkaloid metabolism
AfuA_8g00250	dimethylallyl tryptophan synthase	Fumitermorgin B biosynthesis/ 2ndario metabolism/ alkaloid metabolism
AfuA_8g00220	cytochrome P450	Fumitermorgin B biosynthesis/ 2ndario metabolism/alkaloid metabolism
AfuA_8g00210	dimethylallyl tryptophan synthase FtmPT1	Fumitermorgin B biosynthesis/ 2ndario metabolism/alkaloid metabolism
AfuA_8g00240	cytochrome P450 monooxygenase	Fumitermorgin B biosynthesis/ 2ndario metabolism/alkaloid metabolism
AfuA_8g00230	phytanoyl-CoA dioxygenase	Fumitermorgin B biosynthesis/ 2ndario metabolism/alkaloid metabolism
AfuA_3g13690	pyoverdine/dityrosine biosynthesis family protein	Secondary metabolism/alkaloid metabolism
AfuA_6g09600	zinc metallopeptidase	Secondary metabolism/alkaloid metabolism
AfuA_6g13990	acetyltransferase putative	Secondary metabolism/alkaloid metabolism
AfuA_6g12080	nonribosomal peptide synthase	Secondary metabolism/alkaloid metabolism
AfuA_6g12070	FAD binding domain protein	Secondary metabolism/alkaloid metabolism
AfuA_8g00620	dimethylallyl tryptophan synthase, putative	Secondary metabolism/alkaloid metabolism
AfuA_6g12060	MAK1-like monooxygenase	Secondary metabolism/alkaloid metabolism
AfuA_2g17980	cytochrome P450 monooxygenase	Secondary metabolism/acetate metabolism
AfuA_2g18000	short chain dehydrogenase/oxidoreductase CpoX2	Secondary metabolism/acetate metabolism
AfuA_4g14840	transferase family protein	Acetate ester metabolism
AfuA_2g18010	cytochrome P450 monooxygenase	Secondary metabolism/acetate and alkaloid metabolism
AfuA_1g16590	C2H2 type conidiation transcription factor BrlA	Secondary metabolism/alkaloid metabolism
AfuA_8g00410	methionine aminopeptidase, type II,	Secondary metabolism
AfuA_2g17990	dimethylallyl tryptophan synthase FgaPT1	Secondary metabolism/acetate and alkaloid metabolism
AfuA_2g17960	NADPH dehydrogenase Oye3	Secondary metabolism/acetate metabolism
AfuA_6g12050	nonribosomal peptide synthase	Secondary metabolism/acetate metabolism
AfuA_2g17970	ergot alkaloid biosynthetic protein A	Secondary metabolism/acetate metabolism
AfuA_2g18040	dimethylallyl tryptophan synthase FgaPT2	Secondary metabolism/acetate and alkaloid metabolism
AfuA_2g18060	hypothetical protein	Secondary metabolism/acetate and alkaloid metabolism
AfuA_1g10380	nonribosomal peptide synthase Pes1	Secondary metabolism/acetate and alkaloid metabolism
AfuA_2g18030	catalase Cat	Secondary metabolism/acetate and alkaloid metabolism
AfuA_2g18020	O-acetyltransferase	Secondary metabolism/acetate and alkaloid metabolism
AfuA_8g00510	cytochrome P450 oxidoreductase OrdA-like	Secondary metabolism
AfuA_8g00530	alpha/beta hydrolase	Secondary metabolism
AfuA_8g06420	hypothetical protein	Secondary metabolism
AfuA_8g00550	methyltransferase SirN-like	Secondary metabolism
AfuA_1g11010	short chain oxidoreductase/dehydrogenase	Secondary metabolism
AfuA_2g17530	conidial pigment biosynthesis oxidase Arb2	Secondary metabolism
AfuA_8g00580	glutathione S-transferase	Secondary metabolism
AfuA_8g00560	cytochrome P450 oxidoreductase	Secondary metabolism
AfuA_3g13670	siderochrome-iron transporter	Secondary metabolism
AfuA_3g01400	ABC multidrug transporter	Secondary metabolism
AfuA_2g17540	conidial pigment biosynthesis oxidase Abr1/brown 1	Secondary metabolism
AfuA_3g15290	C6 transcription factor	Secondary metabolism
AfuA_3g13680	hypothetical protein	Secondary metabolism

AfuA_2g17550	conidial pigment biosynthesis protein Ayg1	Secondary metabolism
AfuA_3g12120	fatty acid oxygenase PpoC	Secondary metabolism
AfuA_1g16710	fatty acid elongase (Gig30)	Secondary metabolism
AfuA_3g13640	extracellular serine-rich protein	Secondary metabolism
AfuA_3g13650	integral membrane protein	Secondary metabolism
AfuA_8g00420	C6 finger transcription factor	Secondary metabolism
AfuA_4g00230	oxidoreductase, 2OG-Fe(II) oxygenase	Secondary metabolism
AfuA_4g00210	polyketide synthase	Secondary metabolism
AfuA_3g13660	Ctr copper transporter family protein	Secondary metabolism
AfuA_3g13700	transferase family protein	Secondary metabolism
AfuA_6g13920	adenylate-forming enzyme	Secondary metabolism
AfuA_6g03020	flavin-binding monooxygenase	Secondary metabolism
AfuA_4g00220	metallo-beta-lactamase domain protein	Secondary metabolism
AfuA_8g00400	conserved hypothetical protein	Secondary metabolism
AfuA_6g08540	C6 finger domain protein	Secondary metabolism
AfuA_6g13945	cytochrome P450 monooxygenase	Secondary metabolism
AfuA_4g00225	conserved hypothetical protein	Secondary metabolism
AfuA_8g00390	FAD-dependent monooxygenase (PaxM)	Secondary metabolism
Afua_6g13940	Cytochrome P450 monooxygenase	Secondary metabolism
AfuA_6g12180	conserved hypothetical protein	Secondary metabolism
AfuA_6g13970	FAD-dependent monooxygenase (PaxM)	Secondary metabolism
AfuA_4g14810	cytochrome P450 monooxygenase	Secondary metabolism/ acetate metabolism
Afua_4g14820	transferase family protein	Secondary metabolism/ acetate metabolism
Afua_4g14830	cytochrome P450 monooxygenase	Secondary metabolism/ acetate metabolism
AfuA_4g14780	cytochrome P450 monooxygenase	Secondary metabolism/ acetate metabolism
AfuA_4g14790	cytochrome P450 monooxygenase	Secondary metabolism/ acetate metabolism
AfuA_4g14770	squalene-hopene-cyclase	Secondary metabolism/ acetate metabolism
AfuA_4g14800	short chain dehydrogenase	Secondary metabolism/ acetate metabolism
AfuA_8g00410	methionine aminopeptidase, type II	Secondary metabolism/ acetate metabolism

Supplementary Table 9. Routes involved in sulfate metabolism modulated in *A. fumigatus* during its interaction with *K. pneumoniae*

Protein/Gene	Identifier	Cluster Data	LOG2FC
Cysteine and Methionine Metabolism			
Cysteine synthase	AfuA_5g02180	Transcript	+1.57
Putative cysteine dioxygenase	AfuA_5g14410	Transcript	+3.21
Predicted metacaspase	AfuA_3g14140	Transcript	+1.40
Putative adenosylhomocysteinase- SAH	AfuA_1g10130	Transcript	-1.62
Putative cysteine synthase B	AfuA_4g03930	Transcript	-2.21
Homocysteine S-methyltransferase activity	AfuA_5g01500	Proteomics/Transcript	-0.808/+1.15
Sulfur-related Metabolism			
Predicted bZIP transcription factor MetR	AfuA_4g06530	Transcript	+1.7
predicted carbon-sulfur lyase	AfuA_5g06910	Transcript	+3.03
Putative alpha-ketoglutarate tauD	AfuA_1g17170	Transcript	+4.11
Putative alpha-ketoglutarate tauD	AfuA_6g10720	Transcript	+2.38
Has domain(s) with predicted carbon-sulfur lyase activity	AfuA_8g01638	Transcript	+2.46
Has domain(s) with predicted carbon-sulfur lyase	AfuA_1g03180	Transcript	+2.08
Iron-sulfur cluster-binding protein	AfuA_8g06450	Transcript	+2.06
Sulfuric ester hydrolase	AfuA_6g13440	Transcript	+1.91
Iron-sulfur cluster assembly	AfuA_2g10370	Proteomics/Transcript	+0.437/+1.78
Has domain(s) with predicted carbon-Sulfur lyase activity	AfuA_3g01990	Transcript	+1.71
Has domain(s) with predicted carbon-Sulfur lyase activity	AfuA_7g04120	Transcript	+1.62
Glutamate carboxypeptidase	AfuA_1G03740	Proteomics/Transcript	-0.618/-0.55
Glutathione S transferase	AfuA_1g01370	Transcript	+1.89
Glutathione S transferase	AfuA_2g00590	Transcript	+3.16
Glutathione transferase activity	AfuA_2g15770	Transcript	+1.39
Putative theta class glutathione transferase	AfuA_7g05500	Transcript	+1.90
Predicted glutathione S transferase	AfuA_8g02500	Transcript	+3.03
Predicted glutathione S transferase	AfuA_2g17300	Transcript	+1.43
Bzip developmental regulator-Glio production	AfuA_2g14680	transcript	+0.73
Cytochrome P450 monooxygenase GliF	AfuA_6g09730	Transcript	+5.41
Gliotoxin sulfhydryl oxidase GliT	AfuA_6g09740	Transcript	+5.56
Glioxin transporter GliA	AfuA_6g09710	Transcript	+2.23
Cytochrome P450 monooxygenase GliC	AfuA_6g09670	Transcript	+3.62
Predicted membrane dipeptidase GliJ	AfuA_6g09650	Transcript	+0.75
Non-ribosomal peptide synthetase GliP	AfuA_6g09660	Transcript	+2.42
Zn2Cys6 binuclear transcription factor GliZ	AfuA_6g09630	Transcript	-0,58
Putative pyridoxine biosynthesis protein	AfuA_5g08090	Transcript	+1.55
GNAT-type acetyltransferase/ siderophores biosynthesis	AfuA_1g04450	Transcript	+1.60

+/-numbers referred to up- and down-regulated genes and/or proteins

Supplementary Table 10. Set of oligonucleotides used for qPCR assay

Gene ID	Sequence (5´-3´)	Conc (nM)	Efficiency (%)
AfuA_3g11070 (*pdcA*)	FWD: CACGAAGTAGCGACACTCATT<br>REV: CAGTCGTTCTCCCTCAATCTTC	400	90.8
AfuA_6g07720 (*pck*-acuF)	FWD: CGAGAAGGAGCCCGATATTT<br>REV: GAGGGTGGAATCATCGTAGTT	400	98.4
AfuA_4g11310 (*fbp1*)	FWD: GAGTCAACGGCTTCACCTT<br>REV: CGTCCCAGTACATGCTATTACC	400	80.8
AfuA_4g03960 (*ctf1A*)	FWD: CTCGAACTCGACATTCTCCATC<br>REV: GGTGCGACCCATCTGAAATA	400	89.4
AfuA_1g16590 (C2H2-*blrA*)	FWD: GAAGAGCCACTCCAAGGAAA<br>REV: GCTGTGGGTCTTGGTGTAAT	400	101.6
AfuA_4g06530 (*metR*)	FWD: CACAGAGAAGAACGGCAAATCREV: GATCTTGGACTCGGAACTACTC	400	89.3
AfuA_7g06090 (*pox1*)	FWD: GACTCAGGTGCTCAACTACAA<br>REV: CTCTTGGTACAGACGCATCAT	500	96.7
AfuA_8g02500 (*gst*)	FWD: ATCCCTTCGGAGCCAGTAT<br>REV: CCTCAATCCGCTCAATCTT	400	97.2%
AfuA_8g01670 *(cat2)*	FWD: GAACAACAATTTCCTGGAGTACCT<br>REV: TAGGCGTCGGGAATGATCTC	400	116.4
AfuA_5g09240 *(sod1)*	FWD: ATTAAGCTGATTGGTGCCGAG<br>REV: GTTACCAGTCTTCTTGGACTCCT	400	113.5
AfuA_6g11430 *(aldA)*	FWD: GTCTGACCTTTACGCCACTATC<br>REV: TCTTGCCTTCAACACCCTTC	400	89.2
βtub[1]	FWD: TTCCCAACAACATCCAGACC<br>REV: CGACGGAACATAGCAGTGAA	70	119

Reference

1. Rocha, M.C. *et al. Aspergillus fumigatus* MADS-Box transcription factor rlmA is required for regulation of the cell wall integrity and virulence. *G3 (Bethesda)* **6**, 2983-3002 (2016).
