# Supplementary material for: Integrated multi-omics identifies pathways governing interspecies interaction between A. fumigatus and K. pneumoniae
Source: Commun Biol. 2024 Nov 12;7:1496. doi: 10.1038/s42003-024-07145-x (PMC11557599; doi:10.1038/s42003-024-07145-x)
Supplement: Supplementary file 2 — Description of Additional Supplementary Files [file 42003_2024_7145_MOESM2_ESM.pdf]

Description of Additional Supplementary Files

File name: Supplementary Data 1

Description: Overview of metabolomic analysis.

File name: Supplementary Data 2

Description: The source data behind graphs generated in the paper.
